# miR-345 in Metastatic Colorectal Cancer: A Non-Invasive Biomarker for Clinical Outcome in Non-KRAS Mutant Patients Treated with 3^rd^ Line Cetuximab and Irinotecan

**DOI:** 10.1371/journal.pone.0099886

**Published:** 2014-06-18

**Authors:** Jakob V. Schou, Simona Rossi, Benny V. Jensen, Dorte L. Nielsen, Per Pfeiffer, Estrid Høgdall, Mette Yilmaz, Sabine Tejpar, Mauro Delorenzi, Mogens Kruhøffer, Julia S. Johansen

**Affiliations:** 1 Department of Oncology, Herlev University Hospital, Herlev, Denmark; 2 Bioinformatics Core Facility, Swiss Institute of Bioinformatics, Lausanne, Switzerland; 3 Department of Oncology, Odense University Hospital, Odense, Denmark; 4 Digestive Oncology Unit, University Hospital Gasthuisberg, Leuven, Belgium; 5 Department of Pathology, Herlev University Hospital, Herlev, Denmark; 6 Department of Oncology, Aalborg University Hospital, Aalborg, Denmark; 7 AROS Applied Biotechnology, Aarhus, Denmark; 8 Department of Oncology, University of Lausanne, Lausanne, Switzerland; 9 Ludwig Center for Cancer Research, University of Lausanne, Lausanne, Switzerland; Gustave Roussy, France

## Abstract

**Introduction:**

MicroRNAs (miRNAs) have important regulatory functions in cellular processes and have shown promising potential as prognostic markers for disease outcome in patients with cancer. The aim of the present study was to find miRNA expression profiles in whole blood that were prognostic for overall survival (OS) in patients with metastatic colorectal cancer (mCRC) treated with cetuximab and irinotecan.

**Methods:**

From 138 patients with mCRC in 3^rd^ line therapy with cetuximab and irinotecan in a prospective phase II study, 738 pretreatment miRNAs were isolated and profiled from whole blood using the TaqMan MicroRNA Array v2.0. Mutation status of *KRAS, BRAF, and PI3KCA* was known.

**Results:**

After Bonferroni adjustment, 6 miRNAs: (miR-345, miR-143, miR-34a*, miR-628-5p, miR-886-3p and miR-324-3p), were found associated with short OS. miR-345 was the strongest prognostic miRNA, significant in the full cohort and in the non-*KRAS* mutant population. miR-345, as a continuous variable in the full cohort, resulted in a hazard ratio (HR) of 2.38 per IQR (CI 95%: 1.8–3.1, *P*-value = 2.86e−07, Bonferroni adjusted, univariable analysis) and a HR = 1.75 per IQR (CI 95%: 1.24–2.48, *P*-Wald = 1.45e-03) in the multivariable analysis adjusted for gender, age, KRAS, PI3KCA and performance status. miR-345 was prognostic in progression-free survival (PFS) with a HR = 1.63 per IQR (CI 95%: 1.25–2.114, *P*-Wald = 2.92e-4) in the multivariable analysis. In addition, high miR-345 expression was associated with lack of response to treatment with cetuximab and irinotecan.

**Conclusion:**

We identified miR-345 in whole blood as a potential biomarker for clinical outcome. MiR-345 was a single prognostic biomarker for both OS and PFS in all patients and also in the non-*KRAS* mutant population.

## Introduction

Colorectal cancer (CRC) is the 3^rd^ most common cancer worldwide. Twenty percent of the patients diagnosed with CRC have metastatic disease and an additional 30–35% will develop metastases later during the course of their disease [1], [2].

Cetuximab, an IgG1 monoclonal antibody directed against the Epidermal Growth Factor Receptor (EGFR), has shown efficacy in combination with chemotherapy in patients with metastatic colorectal cancer (mCRC), although not in patients with *KRAS* mutations, [3], [4] which are seen in about 40% of the patients with CRC. However, only a subset of patients with *KRAS* wild type (wt) will benefit from cetuximab. Thus, there is a great need to identify new biomarkers of treatment response to cetuximab in the subgroup of patients with mCRC and *KRAS*.

During the last few years, there has been a rapidly growing interest in microRNAs (miRNAs) as potential new biomarkers in patients with cancer. MiRNAs are small endogenous non-coding RNAs with 19–22 nucleotides regulating gene expression at the posttranscriptional level. To date, more than 2000 human miRNA sequences have been identified, and the number is growing [5]. MiRNAs have important regulatory functions in basic cellular processes and act as oncogenes and tumor-suppressor genes [6]. MiRNAs are involved in cancer predisposition, development and progression through gene deregulation and/or single-nucleotide polymorphism. MiRNAs in a cancer setting can be classified on the basis of their main functions: oncomir, metastamir, apoptomir, hypoxamir and angiomir.

Circulating miRNAs can be easily collected and *seem very stable under harsh conditions, long storage time and after multiple freeze-thaw cycles.* *[7]*, *[8]* *In addition,* a blood sample is more convenient and less invasive for patients than a biopsy.

Besides having a diagnostic potential, [9], [10], [11] certain miRNAs have been shown to be prognostic. Serum miR-141 was suggested as a prognostic biomarker for patients with CRC [12] and serum miR-21 as a diagnostic and prognostic biomarker in CRC.[13] *The aim of the present prospective biomarker study was to evaluate whether profiles of* miRNA in whole blood were prognostic for overall survival and clinical outcome in patients with mCRC before 3^rd^ line treatment with cetuximab and irinotecan.

## Methods

### Patients and Treatment

In a prospective phase II study designed to evaluate the efficacy of cetuximab and irinotecan as 3^rd^ line therapy in patients with mCRC, pretreatment whole blood miRNA expressions were measured in 143 patients. All patients were resistant to 5-FU, oxaliplatin and irinotecan and treated with irinotecan (180 mg/m^2^) and cetuximab (500 mg/m^2^) every second week [14]. Patients were included from three Danish Hospitals from October 2006 to October 2008 and treated until disease progression and followed until death or September 1, 2012.

### Ethics Statement

All patients provided written informed consent, and the study was approved by the Regional Ethics Committee under the Danish National Committee on Health Research Ethics (VEK ref. KA-20060094, www.cvk.sum.dk).

### *KRAS*, *BRAF* and *PI3KCA* Mutation Status

From all patients, formalin fixed paraffin embedded (FFPE) tissue blocks representing the tumor were selected. The FFPE tissues were cut into 3–4 micrometers sections and stained with hematoxylin and eosin (H&E) to evaluate and confirm tumor tissue in the selected tissue block. Briefly, three tissue sections were treated once with xylene, followed with one wash in Ethanol. The pellet was re-suspended in ATL (tissue lysis buffer) buffer and treated with Proteinase-K overnight at 56°C. After inactivation of Proteinase-K by heating the DNA was extracted using the QIAamp DNA Mini Kit.

*KRAS* mutations in codon 12 and 13 were analyzed using the TheraScreen *KRAS* mutation kit (DxS Ltd, Manchester, United Kingdom) which identify 7 somatic *KRAS* mutations. The patients were classified according to whether a mutation was present (*KRAS* mutant, mt) or not (*KRAS* wild type, wt).[15], [16] V600 *BRAF* mutation analyses with a sensitivity of 5% were performed by pyrosequencing (Pyromark Q24) using primers as described by Richman et al [17]. *PIK3CA* mutations were detected using the TheraScreen *PI3KCA* mutation kit (DXS diagnostics), testing for four somatic mutations in exon 9 and in exon 20 of the *PI3KCA* oncogene (p.H104R, p.E542K, p.E545K/D). The mutations p.H104R, p.E542K, p.E545K were detected with a sensitivity of 1% and p.E545D was detected with a sensitivity of 2%.

### Extraction of RNA from PAXgene Blood RNA Tubes

Pretreatment blood samples were collected in PAXgene Blood RNA tubes (Qiagen) and stored at −80°C according to the manufactures instructions. Small RNAs were extracted from the PAXgene Blood RNA tubes in two fractions.[18] These tubes were processed on the BiorobotMDx (Qiagen, Hilden, Germany) using a customized protocol that binds large RNAs and rescues the run-through from the RNA binding plate. The binding condition in the run-through was subsequently modified enabling the miRNA to be purified on an RNeasy-96 plate. The concentration of the small RNA fractions was assessed by absorbance spectrometry on a DTX 880 (Beckman Coulter).

### MiRNA Expression in Whole Blood

The miRNA profiling was performed on TaqMan Array Human MicroRNA cards A and B v2.0 (Applied Biosystems) using the manufactures reagents and instructions. RNA was transcribed into cDNA in two multiplex reactions each containing 3 µl of the small RNA preparation and either Megaplex RT Primer A Pool or Pool B pool and using the TaqMan MicroRNA Reverse Transcription Kit in a total volume of 14 µl. Prior to loading of the arrays a 12 cycle preamplification reaction was performed using 2.5 µl cDNA in a 25 µl reaction. Each of the arrays was loaded with 800 µl Universal PCR MasterMix assay containing 1/40 of the preamplification reaction and run on the 7900 HT Fast Real-Time PCR System.

### MiRNA Expression Analysis

#### Quality assessment

Samples with an absorbance below 1.8 were discarded and values with detection mean cycles greater than 29.5 were considered missing values. We calculated, to assess data quality, the number of detected miRNAs per sample (Figure S1 in file S1) and the mean miRNAs expression per sample (Figure S2 in file S1). Internal controls were analyzed to assess reproducibility.

#### Normalization and filtering

Data were normalized by using the Global Mean Normalization [19] and changes in the expression were calculated using the 2^−ΔCt^ method. Data were standardized before analysis.[20] Only the 138 samples with at least 51% expressed miRNAs were retained. MiRNAs with more than 21% (the cutoff is the estimated level at which the percentage of retained miRNAs reaches a plateau) percent missing values were discarded: 402 out of 768 were retained (372 miRNAs: only part of them CRC specific and 30 control probes: replicates of U6, RNU24, RNU43, RNU44, RNU48 and RNU6B).

Since the samples came from three different Hospitals, we performed Principal Component Analysis to check for bias by hospital. Figure S3 in file S1 shows some differences by center. Data were adjusted by center using the batch-correction algorithm ComBat, [21] from the Bioconductor library sva, [22] see Figure S4 in file S1.

### Statistical Analysis

Data were analyzed with R version 3.0.1. Mann-Whitney (if 2 groups) or Kruskal-Wallis (if more than 2 groups) tests followed by *P*-values adjustment by the Benjamini & Hochberg method (FDR<5%) were performed to determine differentially expressed miRNAs between classes of samples (*KRAS, BRAF, PI3KCA*, double wt, clinical benefit, performance status).

Univariable and multivariable survival analyses were performed using the proportional hazards Cox regression (package “survival” in R). We reported single-test and Bonferroni-adjusted *P*-values based on the number of miRNAs tested in the univariable overall survival (OS) and progression free survival (PFS) regression models. A bootstrap procedure (150 bootstraps as by default for this procedure) from the regression modeling strategy was performed to validate the fitted miRNAs based Cox survival models (R library rms), concordance probabilities were calculated by elaboration of the standard errors (among the other available statistics) between the re-sampled fitted model and the original model at every bootstrap step. The concordance measure represents a probability, it measures how well our model discriminates between different responses, where C = 0.5 implied no predictive ability. In our case, C>0.5 means that that specific miRNAs is a good predictor.

Multivariable survival analysis was performed with the following variables: miRNAs (as continuous variables), gender, *KRAS*, *PI3KCA* and age (dichotomized by median = 63 years). *BRAF* was excluded from multivariable analysis due to the low number of mutated patients with events (n = 2). The expressions of the continuous miRNAs were dichotomized into High and Low risk by the median of the expression values for visualization in the Kaplan-Meier plots. Kaplan-Meier figures were reported for every fitted survival model with at least one statistically significant miRNA. The Kaplan-Meier figures also show Hazard Ratios (HR), confidence intervals (CI) and unadjusted Wald *P*-values from Cox regression models for specific pairwise comparisons of interest. Hazard ratios of continuous variables are presented in interquartile range (IQR) units.

### Target and Pathway Analysis

To identify molecular pathways potentially altered by the expression of single or multiple miRNAs, we used the Diana-mirPath software, [23] a web-based computational tool. The software performs an enrichment analysis of the target genes of multiple miRNAs by comparing them to all KEGG pathways.

The databases used were TarBase (www.microrna.gr/tarbase) or microT-CDS (www.microrna.gr/microT-CDS).

## Results

### Clinical Characteristics and Mutation Status of the Patients

After quality assessment, samples from five patients were discarded. Baseline clinical characteristics and distribution of *KRAS, BRAF,* and *PI3KCA* mutation status of the remaining 138 patients with mCRC included in the present biomarker study are given in Table 1. All patients were treated without knowledge of their mutational status.

**Table 1:** Baseline characteristics of the patients.

	No	%
Age (median)	63 (36–87)	
Male	84	61
Female	54	39
Performance Status		
0	68	49
1	48	35
2	22	16
*KRAS* mutation status		
Wild Type	88	64
Mutant Type	46	33
Undetermined	4	3
*BRAF* mutation status		
Wild Type	123	89
Mutant Type	2	2
Undetermined	13	9
*PI3KCA* mutation status		
Wild Type	105	76
Mutant Type	20	15
Undetermined	13	9

(N = 138).

### miR-345 Was Prognostic for Overall Survival

One hundred and thirty two (95%) patients were dead at time of follow-up (in September 2012). Median overall survival of the patients was 10.6 months (95% CI: 2.1–38.2 months). No difference in OS between the 3 centers was observed.

**Figure 1:**
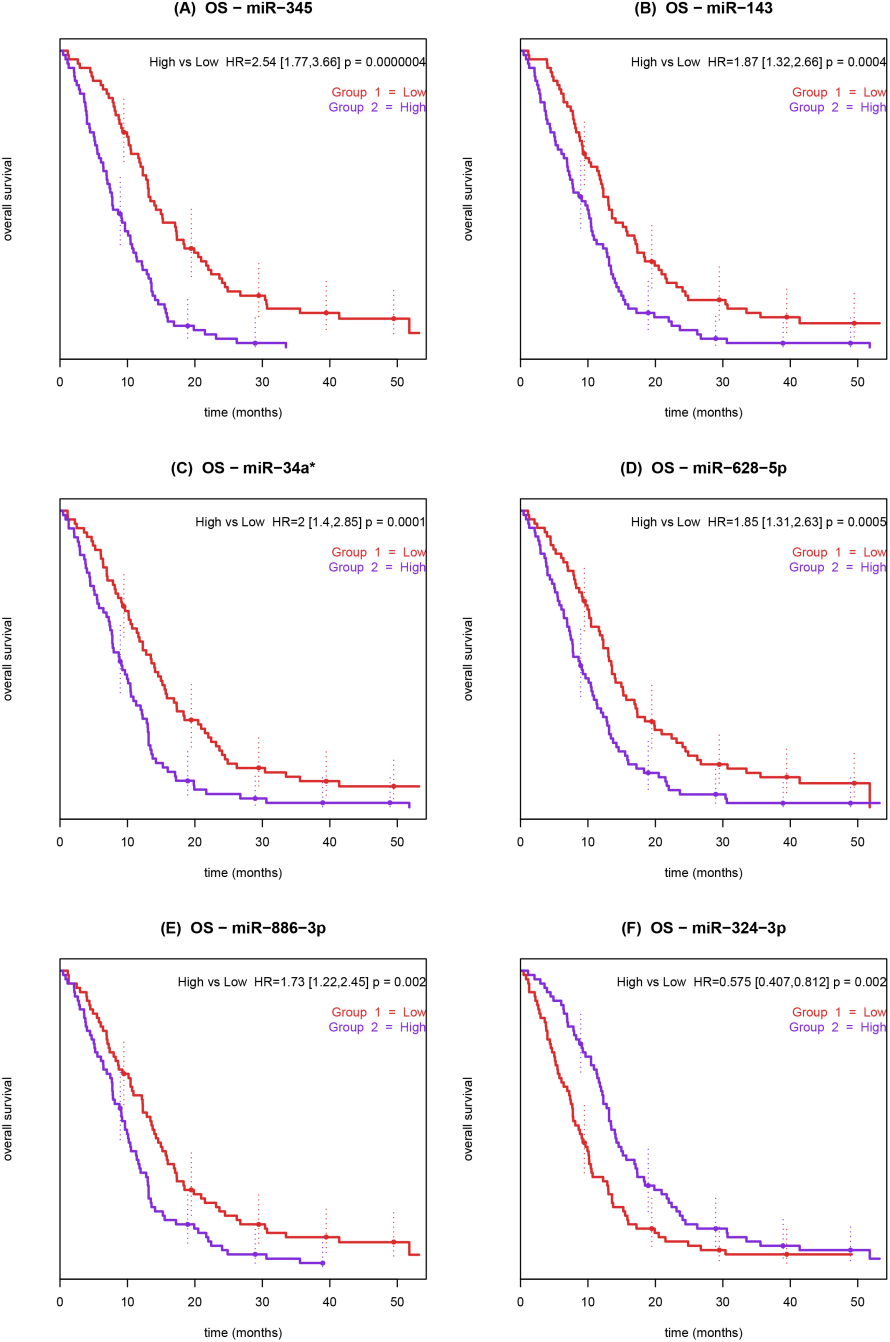
Kaplan-Meier curves showing the association between overall survival and pre-treatment miRNA expression in whole blood from patients with metastatic colorectal cancer treated with 3^rd^ line cetuximab and irinotecan. Patients were dichotomized by the median expression value for miR-345. Low miRNA expression (red) and high miRNA expression (blue). The *P*-value refers to the Wald test.

After adjusting with the Bonferroni method, we found six prognostic miRNAs: high expression of miR-345, miR-143, miR-34a*, miR-628-5p, miR-886-3p and low expression of miR-324-3p were associated with short OS (Table 2). The Kaplan-Meier plots for the six prognostic miRNAs are shown in Figure 1.

**Table 2:** List of prognostic miRNAs for overall survival. Hazard ratios with confidence intervals, Wald *P*-values and Bonferroni adjusted *P*-values are given.

miRNA	HR	lower.95	upper.95	*P*-Wald	*P*-Bonferroni
hsa-miR-345	2.377	1.804	3.132	7.70E-10	2.86E-07
hsa-miR-143	1.989	1.474	2.685	6.95E-06	2.59E-03
hsa-miR-34a*	1.787	1.349	2.369	5.27E-05	1.96E-02
hsa-miR-628-5p	1.693	1.307	2.195	6.84E-05	2.54E-02
hsa-miR-886-3p	1.582	1.255	1.993	1.01E-04	3.76E-02
hsa-miR-324-3p	0.551	0.428	0.71	4.17E-06	1.55E-03

A full list of miRNAs prognostic for OS before Bonferroni correction is provided in Table S1 in file S1;- a total of 64 miRNAs out of 372 were prognostic, 50 with higher expression associated with worse prognosis and 14 with lower expression associated to worse prognosis, none of the controls (U6, RNU24, RNU43, RNU44, RNU48 and RNU6B) were prognostic. A Volcano plot, showing Z score as calculated by Cox regression analysis and an unadjusted p value, is shown in Figure S5 in file S1.

Multivariable survival analysis was performed with the following variables: miR-345, miR-143, miR-34a*, miR-628-5p, miR-886-3p, miR-324-3p, gender, *KRAS, PI3KCA* and age (dichotomized by median = 63 years). Results are reported in Table 3.

**Table 3:** Concordance, Univariable and Multivariable Cox analyses of overall survival and progression-free survival in 138 patients, with metastatic colorectal cancer treated with 3^rd^ line cetuximab and irinotecan according to a miRNAs, gender, age, performance status and *KRAS* and, *PI3KCA* mutation status. miRNAs are tested as continuous variables in interquartile ranges units.

OS	Concordance				Univariate				Multivariate			
	index	lower.95	upper.95	*P*-value(single test)	HR	lower.95	upper.95	Wald *P*-value(single test)	HR	lower.95	upper.95	Wald *P*-value(multiple tests)
hsa-miR-345	0.67	0.63	0.717	4.02E-15	2.377	1.804	3.132	7.70E-10	1.754	1.241	2.479	1.45E-03
hsa-mir-143	0.63	0.575	0.683	2.83E-06	1.989	1.474	2.685	6.95E-06	1.127	0.777	1.635	5.28E-01
hsa-miR-34a*	0.61	0.555	0.66	6.76E-05	1.787	1.349	2.369	5.27E-05	1.191	0.843	1.683	3.23E-01
hsa-miR-628-5p	0.64	0.587	0.685	6.12E-08	1.693	1.307	2.195	6.84E-05	1.049	0.779	1.412	7.54E-01
hsa-miR-886-3p	0.6	0.551	0.658	1.39E-04	1.582	1.255	1.993	1.01E-04	1.251	0.923	1.694	1.49E-01
hsa-miR-324-3p	0.37	0.324	0.419	1.15E-07	0.55	0.428	0.71	4.17E-06	0.7	0.509	0.969	3.16E-02
Gender(female vs. male)	0.52	0.421	0.618	6.93E-01	1.088	0.766	1.545	6.37E-01	1.034	0.688	1.555	8.71E-01
Age median(>63 vs. <63)	0.58	0.482	0.671	1.14E-01	1.269	0.899	1.791	1.75E-01	1.301	0.876	1.931	1.93E-01
*KRAS*(mt. vs. wt)	0.6	0.505	0.7	3.93E-02	1.356	0.936	1.965	1.08E-01	1.666	1.109	2.501	1.39E-02
*PI3KCA*(mt. vs. wt)	0.49	0.35	0.637	9.29E-01	0.89	0.543	1.459	6.44E-01	0.7	0.414	1.17	1.71E-01
Performance status(2 vs. 1 vs. 0)	0.67	0.601	0.743	2.04E-06	1.637	1.288	2.081	5.57E-05	1.406	1.042	1.897	2.59E-02
**PFS**	**Concordance**				**Univariate**				**Multivariate**			
	**index**	**lower.95**	**upper.95**	***P*** **-value** **(single test)**	**HR**	**lower.95**	**upper.95**	**Wald ** ***P*** **-value** **(single test)**	**HR**	**lower.95**	**upper.95**	**Wald ** ***P*** **-value** **(multiple tests)**
hsa-miR-345	0.6	0.545	0.656	4.22E-04	1.7	1.313	2.2	5.53E-05	1.625	1.25	2.114	2.92E-04
Gender(Female vs. Male)	0.5	0.402	0.602	9.65E-01	0.94	0.661	1.325	7.08E-01	0.93	0.626	1.387	7.28E-01
age.median(>63 vs. <63)	0.58	0.487	0.675	9.28E-02	1.384	0.984	1.946	6.18E-02	1.402	0.953	2.063	8.65E-02
*KRAS*(mt. vs. wt)	0.59	0.492	0.681	7.31E-02	1.598	1.099	2.324	1.42E-02	1.633	1.11	2.401	1.28E-02
*PI3KCA*(mt. vs. wt)	0.47	0.331	0.612	6.91E-01	0.9	0.549	1.467	6.65E-01	0.79	0.477	1.293	3.43E-01
Performance status(2 vs. 1 vs. 0)	0.6	0.527	0.681	7.96E-03	1.318	1.05	1.653	1.72E-02	1.334	1.033	1.722	2.72E-02

In OS, miR-345 and miR-324-3p were the only two miRNAs that were statistically significant in both the multivariable analysis and the univariable analysis. Performance status and *KRAS* were also significant factors in the multivariable analysis, although *KRAS* was borderline significant as a univariate factor (HR = 1.36, CI 95%: 0.94–1.97).

### miR-345 as a Single miRNA Marker for Clinical Outcome

**Figure 2:**
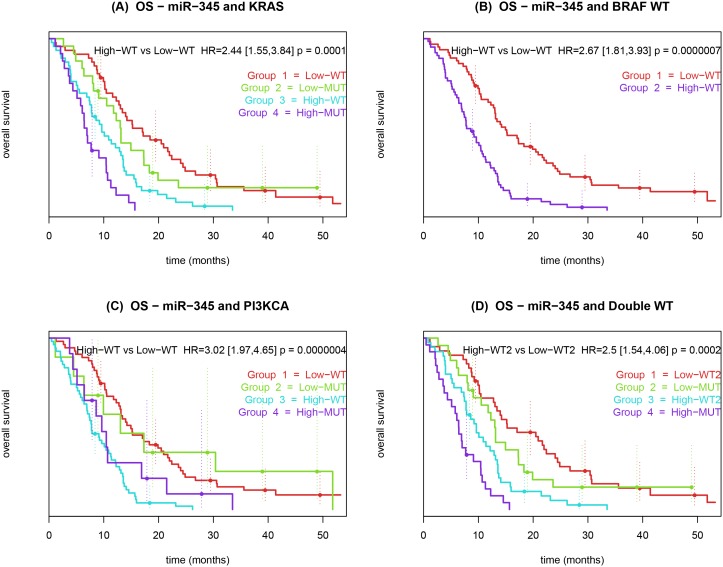
Kaplan-Meier curves showing the association between overall survival and pre-treatment miR-345 expression in whole blood from patients with metastatic colorectal cancer treated with 3^rd^ line cetuximab and irinotecan. (**A**) Patients were dichotomized by *KRAS* mutation status and miR-345. (**B**) Patients were dichotomized by *BRAF* wt mutations status and miR-345. (**C**) Patients were dichotomized by *PI3KCA* mutation status and miR-345. (**D**) Patients were dichotomized by double wt mutation status and miR-345. The *P*-value refers to the Wald test.

**Figure 3:**
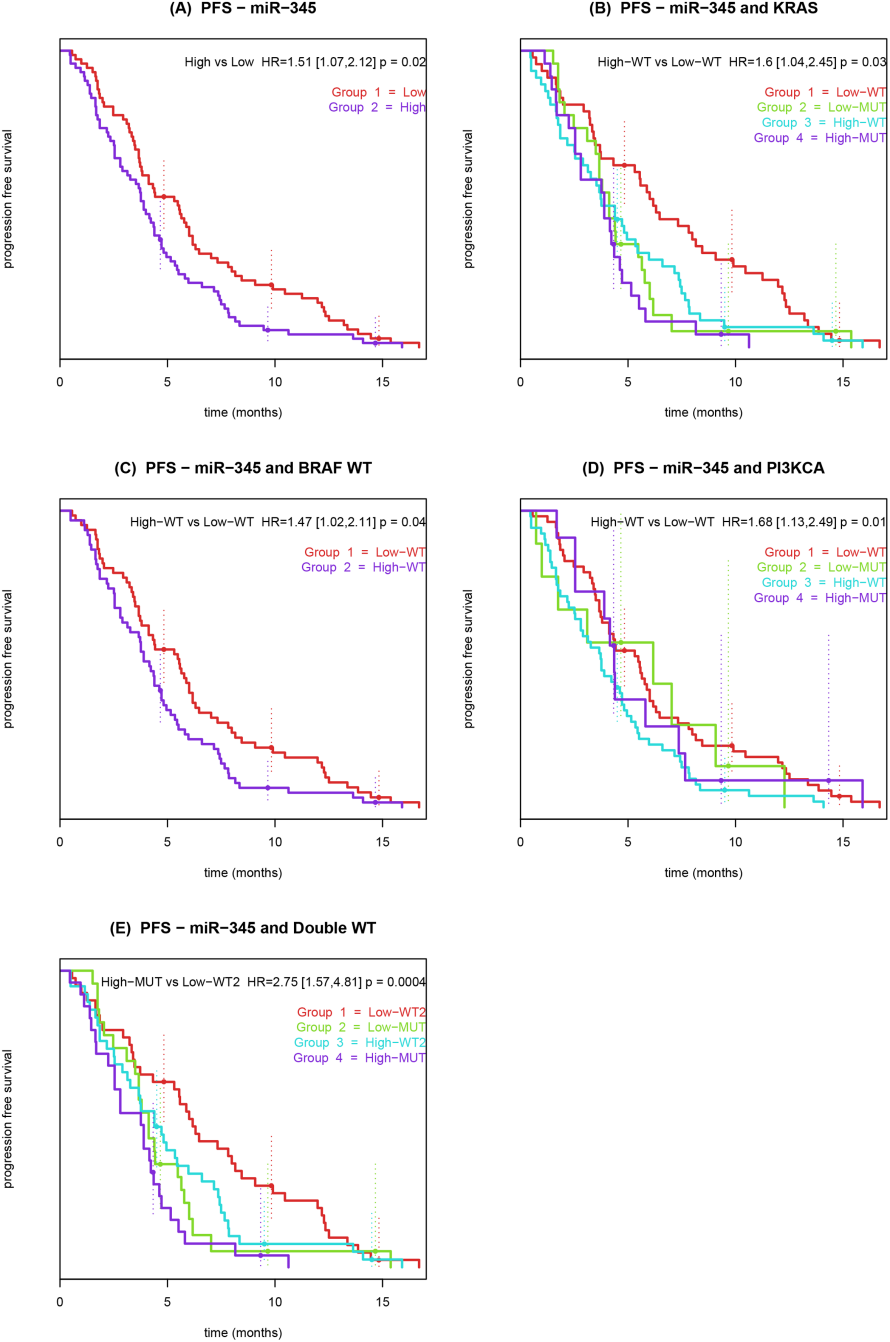
Kaplan-Meier curves showing the association between progression free survival, pre-treatment miR-345 expression in whole blood and mutational status of patients with metastatic colorectal cancer treated with 3^rd^ line cetuximab and irinotecan. Patients were dichotomized by low miR-345 expression (red) and high miR-345 expression (blue). (**A**) Patients were dichotomized by the median expression value for miR-345. (**B**) Patients were dichotomized by *KRAS* mutations status and miR-345. (**C**) Patients were dichotomized by *BRAF* WT mutations status and miR-345. (**D**) Patients were dichotomized by *PI3KCA* mutations status and miR-345. (**E**) Patients were dichotomized by double wt mutation status and miR-345. The *P*-value refers to the Wald test.

miR-345 was also predictive for PFS, HR by IQR = 1.7, (CI 95%: 1.31–2.2, adjusted Bonferroni *P*-value = 0.021). Besides being significant for OS in the entire cohort, miR-345 was statistically significant in all survival models for subgroups of patients with wt of *KRAS*, *BRAF*, *PI3KCA* and double wt for the combinations, *KRAS*+*BRAF* and *KRAS*+*PI3KCA,* see Tables S2–6 in file S1. miR-345 was prognostic for OS in the *BRAF*+*PI3KCA* wt group and in the triple wt group (*BRAF* wt + *KRAS* wt + *PI3KCA* wt) but not in the in the PFS analyses. We compared the available mutational status in combination with the miR-345 expression with regard to OS (Figure 2) and PFS (Figure 3).

The fitted Cox models were validated by resampling procedure. The model fitted on miR-345 and OS showed good concordance, higher than every other analyzed miRNAs in OS (Figure S6 in file S1).

**Figure 4:**
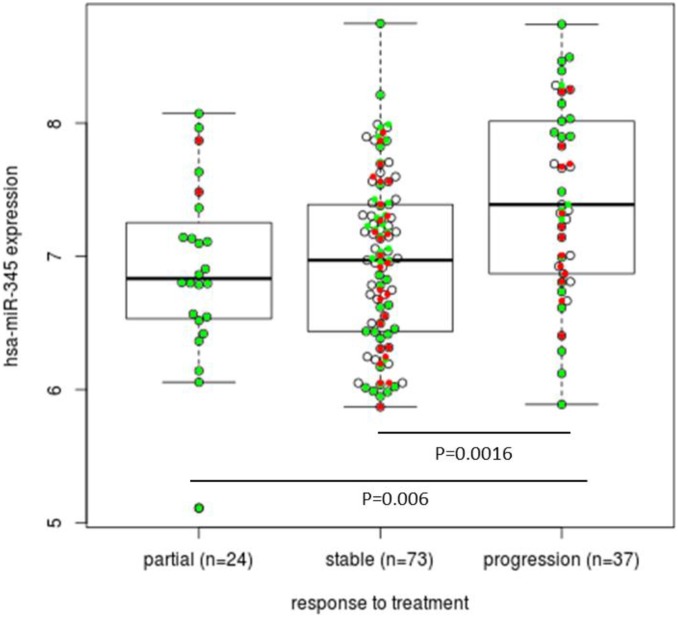
Box plot showing the association between miR-345 expression and response to treatment (partial response, stable disease and progression). Mann-Whitney test; *KRAS* wt patients (green dots), *KRAS* mutated patients (red dots). If we restrict our set of patients to those with partial response (n = 24) and progressive disease (n = 37), a significant relationship between miR-345 expression and response to therapy was obtained with an odds ratio of 5.37, (*P*-value = 0.004 and 95% CI: 1.56–20.94).

We also checked whether miR-345 expression was associated with response to treatment and clinical benefit. We could see that higher expression was associated with lack of response (Figure 4). To analyze this further, we restricted our set of patients to those with a partial response (n = 24) and progressive disease (n = 37) in best overall response. We then compared the number of patients with a lower and higher miR-345 expression according to the median expression of miR-345 and obtained a significant relationship between miR-345 expression and response to therapy with an odds ratio of 5.37 (*P*-value = 0.004; 95% CI: 1.56–20.94).

### Pathway and Target Analysis

To identify molecular pathways associated to the expression of the six miRNAs prognostic for OS (miR-345, miR-143, miR-34a*, miR-628-5p, miR-886-3p, miR-324-3p), we tested KEGG pathways. Among the statistically significant ones, colorectal cancer (KEGG pathway hsa05210) was present due to the predicted interactions between these miRNAs and the following target genes: TCF4, APC, SMAD4, MAPK8, PIK3CG, PIK3CD, DCC, PIK3CG, PIK3CA and MAPK1.

## Discussion

In first and later lines of treatment of patients with mCRC the addition of cetuximab to chemotherapy may be beneficial in patients without mutations in *KRAS* and *BRAF* [24], [25], [26]. However, recent studies investigating cetuximab in first line mCRC, could not confirm a benefit in PFS and OS in *KRAS* wt patients.[27], [28]. There is a need for more precise biomarkers or panels of biomarkers. The present prospective biomarker study was conducted to identify prognostic miRNAs in whole blood from patients with mCRC treated with 3^rd^ line cetuximab and irinotecan.

Our study suggests a potential role of circulating miRNAs to serve as biomarkers for clinical outcome in patients with mCRC. We found that miR-345 was prognostic for OS in all our patients with mCRC and in the *KRAS* wt patients, with high expression associated with shorter survival. Multivariable analysis demonstrated that miR-345 was a strong factor that added prognostic information to the other available clinicopathologic and mutation variables. Tumor burden is a combination of tumor size and number of metastatic sites. Data regarding tumor size was not available to us, and therefore tumor burden could not be included in the multivariable analysis. We did, however, include a representative list of covariates which are regarded as prognostic factors for patients with mCRC treated with cetuximab.

miR-345 was statistically significant for all wt subgroups analyzed. Since all patients were treated with cetuximab, we cannot deduce whether miR-345 was a prognostic biomarker (independent of treatment the patients received) or a biomarker predicting sensitivity to treatment with cetuximab and irinotecan, but we found that its higher expression was associated with lack of response to therapy. In the isolated group of responders versus non-responders, a high miR-345 expression was significantly associated with a 5 times risk of progressive disease as best overall response. These results are novel findings and suggest that this miRNA is prognostic and may be a potential biomarker for response in patients with mCRC treated with cetuximab and irinotecan.

Circulating miR-345 has to our knowledge, not previously been described in connection with CRC, but Tang and colleagues reported that a low level of miR-345 in CRC tissue is associated with lymph node metastases, worse histological type and is up-regulated in colorectal cancer cell lines after treatment with 5-aza-dc [29]. In addition, DNA-methylation levels in the promoter region of miR-345 are higher in CRC tissue compared to normal colon tissue and corresponding non-cancerous tissue, suggesting that this miRNA is a methylation sensitive tumor suppressor [29].

In tissue samples, miR-345 is prognostic in patients with breast cancer [30]. Although validation in external cohorts was not statistically significant, miR-345 was the only miRNA to remain prognostic in a multivariable model, accounting for key clinical parameters [30].

It is not known why there is a discrepancy between miRNA expression in tissue and blood from patients with CRC. Few studies have compared miRNA expression in corresponding tissue and blood samples. Little overlap is seen between miRNAs differentiating lung cancer samples from normal lung tissue samples and plasma miRNAs differentiating patients with aggressive vs. non-aggressive lung cancer.[31] miR-345 was found decreased in CRC tissue in a relatively small study of 26 CRC tissue samples in which only 5 patients had metastatic disease. [29] A low miR-345 concentration in predominantly localized CRC tissue may not be comparable to high concentration of circulating miR-345 in metastatic CRC. The same miRNA may be tumor-related in different ways, depending on, if it is found in the tumor or as a circulating miRNA.

miR-143 is down- regulated in CRC tumor samples as compared to normal tissue.[32], [33], [34] *KRAS* may be a target for miR-143[35] and levels of miR-143 expression in CRC tumour samples are a prognostic biomarker in *KRAS* wt patients but not a predictive marker for anti-EGFR treatment.[36] Circulating miR-143 has been tested as diagnostic marker in CRC patients, but is not significantly deregulated compared to normal controls.[37] miR-324-3p has not been described in CRC, but is differently expressed in plasma from patients with breast cancer compared to healthy controls.[38].

The strength of our study was the relatively large sample size, bootstrap procedure (resampling validation of the Cox regression model), and a strict statistical approach using only miRNAs adjusted in accordance with the Bonferroni correction. The next step will be to validate these significant miRNAs in whole blood from another cohort of patients with mCRC treated with cetuximab and irinotecan. At present no such samples are available from completed phase III studies.

No studies have investigated whether miRNA expressions in whole blood have a potential as prognostic or predictive biomarkers in patients with mCRC treated with cetuximab and irinotecan. Most studies investigating circulating miRNAs have used plasma or serum.[39], [40] miRNA expression profiles in whole blood collected in PAXgene RNA tubes have been described in patients with hematologic diseases, pancreatic and lung cancer [41], [42] and have advantages compared to miRNA in serum/plasma due to higher yield [43] of miRNAs and fewer methodological problems [44]. MiRNAs found in peripheral whole blood can originate from cancer cells but also from non-cancerous cell such as leucocytes, thrombocytes and monocytes. They can be in the blood stream bound to proteins like AGO [45] and HDL [46] or packed in exosomes.[47] The prognostic miRNAs, found in our group of patients, may be due to tumor progression, an inflammatory response or as a response to organ failure.

Recently, Shen and colleagues reported, that EGFR was involved in the suppression of specific miRNA maturation in response to hypoxic stress.[48] The proposed mechanism was that an EGFR through phosphorylation of AGO2, caused a reduction of DICER/AGO2 binding [48] hereby inhibiting the maturation of pre-miRNAs with long-loop configuration. Inhibition of EGFR may also cause intracellular miRNA regulation and transport some of the altered miRNAs into the blood stream, thereby acting as a circulating response to EGFR actions.

## Conclusion

We identified six circulating miRNAs in whole blood as prognostic markers for OS. miR-345 was a single biomarker for OS in all patients and in subgroups of patients with *KRAS* wt and *KRAS+BRAF* double wt. Furthermore, miR-345 was significantly associated with PFS and best overall response and may be a potential biomarker for clinical outcome in patient with mCRC treated with 3^rd^ line cetuximab and irinotecan.

## Supporting Information

File S1 Contains the following files: Figure S1, Figure S2, Figure S3, Figure S4, Table S1, Table S2, Table S3, Table S4, Table S5, Table S6, Figure S5, Figure S6.
(DOCX)
