# Supplementary material for: miR-345 in Metastatic Colorectal Cancer: A Non-Invasive Biomarker for Clinical Outcome in Non-KRAS Mutant Patients Treated with 3rd Line Cetuximab and Irinotecan
Source: PLoS One. 2014 Jun 18;9(6):e99886. doi: 10.1371/journal.pone.0099886 (PMC4062472; doi:10.1371/journal.pone.0099886)

**miR-345 in metastatic colorectal cancer: A non-invasive biomarker for clinical outcome in non-KRAS mutant patients**

Contents

[Figure S1 2](#_Toc387353452)

[Figure S2 3](#_Toc387353453)

[Figure S3 4](#_Toc387353454)

[Figure S4 5](#_Toc387353455)

[Table S1 6](#_Toc387353456)

[Table S2 8](#_Toc387353457)

[Table S3 10](#_Toc387353458)

[Table S4 12](#_Toc387353459)

[Table S5 14](#_Toc387353460)

[Table S6 17](#_Toc387353461)

[Figure S5. 19](#_Toc387353462)

[Figure S6 20](#_Toc387353463)

# Figure S1

Figure S1. Number of miRNAs detected per sample. Dotted blue line indicates the population mean +/- two times the standards deviation.

# Figure S2

Figure S2. Mean miRNA expression (Ct) per sample. Dotted blue line indicates the population mean +/- two times the standards deviation.

# Figure S3

Figure S3. Two component PCA (principal component analysis) with increasing number of features to provide information concerning the features (miRNAs) that have the stronger effect. PCA analysis was performed on batch effect un-corrected data.

# Figure S4

Figure S4. Two component principal component analysis (PCA), with increasing number of features to provide information concerning the features (miRNAs) that have the stronger effect. PCA analysis was performed on batch effect corrected data (by ComBat)

# Table S1

Table S1. Prognostic miRNAs in OS in patients with metastatic colorectal cancer. In bold those with Bonferroni *P*-value < 0.05. *est: estimate, se: standard error, P Wald: P-value from Wald test, P Bonferroni: P-value adjusted by the Bonferroni method.*

| **miRNA** | **est** | **se** | **Z** | ***P* Wald** | ***P* Bonferroni** |
| --- | --- | --- | --- | --- | --- |
| **hsa-miR-345** | **0.617383** | **0.10037** | **6.151094** | **7.70E-10** | **2.86E-07** |
| **hsa-miR-143** | **0.484088** | **0.107692** | **4.495124** | **6.95E-06** | **0.002586** |
| **hsa-miR-34a*** | **0.390936** | **0.096686** | **4.043368** | **5.27E-05** | **0.0196** |
| **hsa-miR-628-5p** | **0.474125** | **0.119075** | **3.981746** | **6.84E-05** | **0.025449** |
| **hsa-miR-886-3p** | **0.364262** | **0.093689** | **3.887977** | **0.000101** | **0.037603** |
| hsa-miR-943 | 0.383996 | 0.102731 | 3.737895 | 0.000186 | 0.069031 |
| hsa-miR-936 | 0.404337 | 0.108349 | 3.731815 | 0.00019 | 0.070719 |
| hsa-miR-145 | 0.372145 | 0.101122 | 3.680148 | 0.000233 | 0.086713 |
| hsa-miR-29a | 0.347678 | 0.09559 | 3.637169 | 0.000276 | 0.102542 |
| hsa-miR-210 | 0.349192 | 0.096952 | 3.601686 | 0.000316 | 0.117612 |
| hsa-miR-7 | 0.389193 | 0.108836 | 3.575954 | 0.000349 | 0.129811 |
| hsa-miR-30a* | 0.305863 | 0.085889 | 3.561169 | 0.000369 | 0.137345 |
| hsa-miR-146b-5p | 0.326697 | 0.098804 | 3.306527 | 0.000945 | 0.351392 |
| hsa-miR-145* | 0.306975 | 0.093077 | 3.298062 | 0.000974 | 0.36216 |
| hsa-miR-330-3p | 0.299244 | 0.091492 | 3.270711 | 0.001073 | 0.399072 |
| hsa-miR-194 | 0.312349 | 0.096104 | 3.250112 | 0.001154 | 0.429138 |
| hsa-miR-744* | 0.311809 | 0.096158 | 3.242662 | 0.001184 | 0.440518 |
| hsa-miR-19b-1* | 0.28814 | 0.089335 | 3.225381 | 0.001258 | 0.467994 |
| hsa-miR-26a-1* | 0.351785 | 0.109479 | 3.21325 | 0.001312 | 0.48822 |
| hsa-miR-197 | 0.317925 | 0.099058 | 3.209471 | 0.00133 | 0.494684 |
| hsa-miR-192 | 0.307245 | 0.0959 | 3.203817 | 0.001356 | 0.504501 |
| hsa-miR-26b* | 0.31429 | 0.099173 | 3.169113 | 0.001529 | 0.568806 |
| hsa-miR-34a | 0.284774 | 0.09096 | 3.130758 | 0.001744 | 0.648604 |
| hsa-miR-339-3p | 0.308396 | 0.09944 | 3.101336 | 0.001926 | 0.716656 |
| hsa-miR-625* | 0.29692 | 0.095767 | 3.100438 | 0.001932 | 0.718834 |
| hsa-miR-149* | 0.317064 | 0.104767 | 3.026368 | 0.002475 | 0.920741 |
| hsa-miR-361-5p | 0.288511 | 0.096309 | 2.99569 | 0.002738 | 1 |
| hsa-miR-627 | 0.289534 | 0.101575 | 2.85044 | 0.004366 | 1 |
| hsa-miR-650 | 0.283184 | 0.099523 | 2.8454 | 0.004436 | 1 |
| hsa-miR-139-3p | 0.290952 | 0.102748 | 2.831712 | 0.00463 | 1 |
| hsa-miR-215 | 0.265115 | 0.094952 | 2.792101 | 0.005237 | 1 |
| hsa-miR-33a* | 0.291903 | 0.104605 | 2.790531 | 0.005262 | 1 |
| hsa-miR-338-3p | 0.285182 | 0.10228 | 2.788238 | 0.0053 | 1 |
| hsa-miR-362-3p | 0.24619 | 0.088418 | 2.784387 | 0.005363 | 1 |
| hsa-miR-330-5p | 0.297319 | 0.107417 | 2.767892 | 0.005642 | 1 |
| hsa-miR-503 | 0.279587 | 0.102528 | 2.726932 | 0.006393 | 1 |
| hsa-miR-148a* | 0.270829 | 0.099473 | 2.722624 | 0.006477 | 1 |
| hsa-let-7f-2* | 0.288637 | 0.107191 | 2.692734 | 0.007087 | 1 |
| hsa-miR-183* | 0.267835 | 0.099613 | 2.688758 | 0.007172 | 1 |
| hsa-miR-182* | 0.266871 | 0.099409 | 2.684581 | 0.007262 | 1 |
| hsa-miR-511 | 0.241904 | 0.090705 | 2.666918 | 0.007655 | 1 |
| hsa-miR-616 | 0.242217 | 0.091322 | 2.652355 | 0.007993 | 1 |
| hsa-miR-505* | 0.248359 | 0.093964 | 2.64313 | 0.008214 | 1 |
| hsa-miR-542-5p | 0.285582 | 0.108924 | 2.621856 | 0.008745 | 1 |
| hsa-miR-331-5p | 0.245333 | 0.094214 | 2.603991 | 0.009215 | 1 |
| hsa-miR-221* | 0.234091 | 0.09006 | 2.599279 | 0.009342 | 1 |
| hsa-miR-886-5p | 0.229424 | 0.088295 | 2.598383 | 0.009366 | 1 |
| hsa-miR-598 | 0.26487 | 0.103668 | 2.55498 | 0.010619 | 1 |
| hsa-miR-889 | 0.261236 | 0.102414 | 2.55079 | 0.010748 | 1 |
| hsa-miR-192* | 0.256393 | 0.100737 | 2.545169 | 0.010922 | 1 |
| **hsa-miR-324-3p** | **-0.45312** | **0.098447** | **-4.60268** | **4.17E-06** | **0.001552** |
| hsa-miR-342-5p | -0.326 | 0.089592 | -3.63871 | 0.000274 | 0.101932 |
| hsa-miR-31 | -0.32546 | 0.089662 | -3.62982 | 0.000284 | 0.105507 |
| hsa-miR-92a-1* | -0.34763 | 0.103535 | -3.35763 | 0.000786 | 0.292444 |
| hsa-miR-532-3p | -0.32909 | 0.111392 | -2.95434 | 0.003133 | 1 |
| hsa-miR-589* | -0.25321 | 0.091397 | -2.77038 | 0.005599 | 1 |
| hsa-miR-652 | -0.24508 | 0.093793 | -2.61297 | 0.008976 | 1 |
| hsa-miR-150 | -0.21978 | 0.086959 | -2.52736 | 0.011492 | 1 |
| hsa-miR-144* | -0.19725 | 0.085643 | -2.30317 | 0.021269 | 1 |
| hsa-miR-328 | -0.22996 | 0.102765 | -2.23769 | 0.025241 | 1 |
| hsa-miR-484 | -0.18215 | 0.08841 | -2.06024 | 0.039376 | 1 |
| hsa-miR-423-5p | -0.19149 | 0.094896 | -2.01786 | 0.043606 | 1 |
| hsa-let-7e | -0.18129 | 0.091216 | -1.98743 | 0.046875 | 1 |
| hsa-miR-191 | -0.17007 | 0.086339 | -1.9698 | 0.048861 | 1 |

# Table S2

Table S2. miRNAs prognostic for OS in the subset of patients KRAS wt. In bold those with Bonferroni *P*-value < 0.05. *est: estimate, se: standard error, P Wald: P-value from Wald test, P Bonferroni: P-value adjusted by the Bonferroni method, P*-FDR**:** *P-value from FDR (False Discovery Rate).*

| miRNA | **est** | **se** | **Z** | ***P* Wald** | ***P* Bonferroni** | ***P*-FDR** |
| --- | --- | --- | --- | --- | --- | --- |
| **hsa-miR-345** | **0.633117** | **0.132138** | **4.791338** | **1.66E-06** | **0.000616** | **0.000616** |
| **hsa-miR-628-5p** | **0.595551** | **0.153673** | **3.875435** | **0.000106** | **0.039594** | **0.01332** |
| **hsa-miR-943** | **0.536317** | **0.138469** | **3.873191** | **0.000107** | **0.03996** | **0.01332** |
| hsa-miR-34a* | 0.432299 | 0.122322 | 3.534097 | 0.000409 | 0.152211 | 0.032861 |
| hsa-miR-210 | 0.420574 | 0.120483 | 3.490734 | 0.000482 | 0.179191 | 0.032861 |
| hsa-miR-26b* | 0.483007 | 0.139392 | 3.465112 | 0.00053 | 0.197164 | 0.032861 |
| hsa-miR-143 | 0.474661 | 0.139563 | 3.401045 | 0.000671 | 0.249719 | 0.035674 |
| hsa-miR-886-3p | 0.398756 | 0.120131 | 3.319337 | 0.000902 | 0.335661 | 0.041958 |
| hsa-miR-7 | 0.457786 | 0.143812 | 3.183221 | 0.001456 | 0.541804 | 0.051114 |
| hsa-miR-19b-1* | 0.355667 | 0.11211 | 3.172478 | 0.001511 | 0.562256 | 0.051114 |
| hsa-miR-145 | 0.384105 | 0.126644 | 3.03294 | 0.002422 | 0.900922 | 0.075077 |
| hsa-miR-625* | 0.355213 | 0.118536 | 2.996663 | 0.00273 | 1 | 0.078106 |
| hsa-miR-936 | 0.396158 | 0.133837 | 2.960005 | 0.003076 | 1 | 0.081743 |
| hsa-miR-330-3p | 0.342702 | 0.118327 | 2.896237 | 0.003777 | 1 | 0.085054 |
| hsa-let-7g | 0.360714 | 0.126255 | 2.857021 | 0.004276 | 1 | 0.085054 |
| hsa-miR-338-3p | 0.360153 | 0.126297 | 2.851626 | 0.00435 | 1 | 0.085054 |
| hsa-miR-26a-1* | 0.394573 | 0.138746 | 2.843851 | 0.004457 | 1 | 0.085054 |
| hsa-miR-744* | 0.366429 | 0.131515 | 2.786218 | 0.005333 | 1 | 0.091877 |
| hsa-miR-194 | 0.342448 | 0.123177 | 2.780136 | 0.005434 | 1 | 0.091877 |
| hsa-miR-145* | 0.321629 | 0.11926 | 2.696866 | 0.007 | 1 | 0.110162 |
| hsa-miR-29a | 0.31673 | 0.118057 | 2.682848 | 0.0073 | 1 | 0.110162 |
| hsa-miR-889 | 0.362271 | 0.136375 | 2.656428 | 0.007897 | 1 | 0.110162 |
| hsa-miR-197 | 0.332667 | 0.125335 | 2.654229 | 0.007949 | 1 | 0.110162 |
| hsa-miR-589 | 0.350293 | 0.132074 | 2.652253 | 0.007996 | 1 | 0.110162 |
| hsa-miR-27a* | 0.351491 | 0.135326 | 2.597369 | 0.009394 | 1 | 0.121895 |
| hsa-miR-627 | 0.318898 | 0.123239 | 2.587643 | 0.009664 | 1 | 0.121895 |
| hsa-miR-192 | 0.287207 | 0.111245 | 2.581744 | 0.00983 | 1 | 0.121895 |
| hsa-miR-616 | 0.317403 | 0.124309 | 2.553343 | 0.010669 | 1 | 0.124099 |
| hsa-miR-30a* | 0.26831 | 0.10509 | 2.553156 | 0.010675 | 1 | 0.124099 |
| hsa-miR-339-3p | 0.298852 | 0.118157 | 2.529285 | 0.01143 | 1 | 0.128842 |
| hsa-miR-186* | 0.271711 | 0.109031 | 2.492056 | 0.012701 | 1 | 0.130037 |
| hsa-miR-330-5p | 0.341961 | 0.137386 | 2.489059 | 0.012808 | 1 | 0.130037 |
| hsa-miR-221* | 0.287281 | 0.116586 | 2.464112 | 0.013735 | 1 | 0.130037 |
| hsa-miR-550 | 0.282421 | 0.114623 | 2.463921 | 0.013743 | 1 | 0.130037 |
| hsa-miR-29c | 0.330158 | 0.134452 | 2.455587 | 0.014065 | 1 | 0.130037 |
| hsa-miR-33a* | 0.33538 | 0.13674 | 2.452688 | 0.014179 | 1 | 0.130037 |
| hsa-miR-148a* | 0.315728 | 0.12996 | 2.429424 | 0.015123 | 1 | 0.130037 |
| hsa-miR-10b | 0.343641 | 0.141627 | 2.426383 | 0.01525 | 1 | 0.130037 |
| hsa-miR-192* | 0.315998 | 0.130363 | 2.423994 | 0.015351 | 1 | 0.130037 |
| hsa-miR-551a | 0.294176 | 0.121395 | 2.423288 | 0.015381 | 1 | 0.130037 |
| hsa-miR-149* | 0.313493 | 0.132568 | 2.364769 | 0.018041 | 1 | 0.147197 |
| hsa-miR-30d* | 0.347816 | 0.147287 | 2.361487 | 0.018202 | 1 | 0.147197 |
| hsa-miR-212 | 0.260953 | 0.112218 | 2.325409 | 0.02005 | 1 | 0.158694 |
| hsa-miR-183* | 0.29554 | 0.127872 | 2.311217 | 0.020821 | 1 | 0.159105 |
| hsa-miR-181c* | 0.263337 | 0.114439 | 2.301116 | 0.021385 | 1 | 0.159105 |
| hsa-miR-650 | 0.286682 | 0.125337 | 2.287292 | 0.022179 | 1 | 0.160453 |
| hsa-miR-215 | 0.266305 | 0.116927 | 2.277537 | 0.022754 | 1 | 0.160453 |
| hsa-miR-146b-5p | 0.280137 | 0.124099 | 2.257364 | 0.023985 | 1 | 0.165232 |
| hsa-miR-425* | 0.348895 | 0.155687 | 2.241004 | 0.025026 | 1 | 0.169266 |
| hsa-miR-935 | 0.249464 | 0.112528 | 2.216901 | 0.02663 | 1 | 0.176898 |
| hsa-miR-938 | 0.298292 | 0.136665 | 2.18265 | 0.029062 | 1 | 0.189665 |
| hsa-miR-181a | 0.266809 | 0.123113 | 2.167188 | 0.030221 | 1 | 0.190156 |
| hsa-miR-34a | 0.259116 | 0.119772 | 2.163412 | 0.03051 | 1 | 0.190156 |
| hsa-miR-182* | 0.263114 | 0.121738 | 2.161324 | 0.03067 | 1 | 0.190156 |
| hsa-miR-28-5p | 0.242573 | 0.113427 | 2.138588 | 0.032469 | 1 | 0.193973 |
| hsa-miR-362-3p | 0.248798 | 0.116731 | 2.131389 | 0.033057 | 1 | 0.193973 |
| hsa-miR-139-5p | 0.286544 | 0.134468 | 2.130949 | 0.033093 | 1 | 0.193973 |
| hsa-miR-148b* | 0.266079 | 0.12543 | 2.121341 | 0.033893 | 1 | 0.193973 |
| hsa-miR-616* | 0.243767 | 0.117179 | 2.080291 | 0.037499 | 1 | 0.208202 |
| hsa-miR-542-5p | 0.28328 | 0.136983 | 2.068002 | 0.03864 | 1 | 0.2107 |
| hsa-let-7f-2* | 0.281981 | 0.136663 | 2.063328 | 0.039081 | 1 | 0.2107 |
| hsa-miR-106b* | 0.222024 | 0.108002 | 2.05575 | 0.039807 | 1 | 0.211544 |
| hsa-miR-505* | 0.239021 | 0.117489 | 2.034411 | 0.04191 | 1 | 0.214014 |
| hsa-miR-139-3p | 0.26114 | 0.128416 | 2.033545 | 0.041997 | 1 | 0.214014 |
| hsa-miR-7 | 0.270046 | 0.134252 | 2.011495 | 0.044273 | 1 | 0.221027 |
| hsa-miR-219-1-3p | 0.26574 | 0.134549 | 1.975053 | 0.048262 | 1 | 0.233538 |
| hsa-miR-146a | 0.255111 | 0.129211 | 1.974369 | 0.04834 | 1 | 0.233538 |
| hsa-miR-95 | -0.25008 | 0.124496 | -2.00877 | 0.044562 | 1 | 0.221027 |
| hsa-miR-144* | -0.21267 | 0.103751 | -2.04977 | 0.040387 | 1 | 0.211603 |
| hsa-let-7d* | -0.24575 | 0.117958 | -2.08336 | 0.037218 | 1 | 0.208202 |
| hsa-miR-296-5p | -0.27046 | 0.127476 | -2.12168 | 0.033865 | 1 | 0.193973 |
| hsa-miR-150 | -0.25151 | 0.110518 | -2.27576 | 0.02286 | 1 | 0.160453 |
| hsa-miR-532-3p | -0.31511 | 0.136631 | -2.30632 | 0.021093 | 1 | 0.159105 |
| hsa-miR-342-5p | -0.25239 | 0.101305 | -2.49143 | 0.012723 | 1 | 0.130037 |
| hsa-miR-31 | -0.32475 | 0.114521 | -2.83568 | 0.004573 | 1 | 0.085054 |
| hsa-miR-92a-1* | -0.38151 | 0.130353 | -2.92677 | 0.003425 | 1 | 0.08494 |
| hsa-miR-324-3p | -0.38668 | 0.119418 | -3.23801 | 0.001204 | 0.447759 | 0.049751 |

# Table S3

Table S3 miRNAs prognostic for overall survival in patients with metastatic colorectal cancer in the subset of patients BRAF wt. In bold those with Bonferroni *P*-value < 0.05. *est: estimate, se: standard error, P Wald: P-value from Wald test, P Bonferroni: P-value adjusted by the Bonferroni method, P*-FDR**:** *P-value from FDR (False Discovery Rate).*

| miRNA | **est** | | **se** | | **Z** | ***P* Wald** | | ***P* Bonferroni** | | ***P*-FDR** |  |
| --- | --- | --- | --- | --- | --- | --- | --- | --- | --- | --- | --- |
| **hsa-miR-345** | | **0.565518** | | **0.103533** | **5.462206** | | **4.70E-08** | **1.75E-05** | **1.75E-05** | | |
| **hsa-miR-143** | | **0.527159** | | **0.117495** | **4.486642** | | **7.24E-06** | **0.002692** | **0.001346** | | |
| **hsa-miR-29a** | | **0.401176** | | **0.103198** | **3.887452** | | **0.000101** | **0.037684** | **0.008076** | | |
| **hsa-miR-34a*** | | **0.395837** | | **0.102267** | **3.870631** | | **0.000109** | **0.040382** | **0.008076** | | |
| hsa-miR-936 | | 0.436384 | | 0.115113 | 3.790904 | | 0.00015 | 0.055837 | 0.009306 | | |
| hsa-miR-145 | | 0.374561 | | 0.104882 | 3.571254 | | 0.000355 | 0.132163 | 0.01652 | | |
| hsa-miR-943 | | 0.377198 | | 0.108551 | 3.474839 | | 0.000511 | 0.190152 | 0.017524 | | |
| hsa-miR-145* | | 0.345403 | | 0.099506 | 3.471169 | | 0.000518 | 0.192769 | 0.017524 | | |
| hsa-miR-628-5p | | 0.432019 | | 0.127141 | 3.39796 | | 0.000679 | 0.252552 | 0.021046 | | |
| hsa-miR-194 | | 0.350915 | | 0.104367 | 3.362315 | | 0.000773 | 0.287526 | 0.022117 | | |
| hsa-miR-34a | | 0.311922 | | 0.094151 | 3.31298 | | 0.000923 | 0.343385 | 0.024527 | | |
| hsa-miR-30a* | | 0.294835 | | 0.090229 | 3.267639 | | 0.001084 | 0.403428 | 0.025038 | | |
| hsa-miR-192 | | 0.338913 | | 0.104009 | 3.258503 | | 0.00112 | 0.416647 | 0.025038 | | |
| hsa-miR-886-3p | | 0.315207 | | 0.096914 | 3.252436 | | 0.001144 | 0.425644 | 0.025038 | | |
| hsa-miR-330-3p | | 0.302382 | | 0.095648 | 3.1614 | | 0.00157 | 0.584087 | 0.031469 | | |
| hsa-miR-7 | | 0.358983 | | 0.114937 | 3.123312 | | 0.001788 | 0.665241 | 0.031469 | | |
| hsa-miR-210 | | 0.314496 | | 0.100756 | 3.121369 | | 0.0018 | 0.669645 | 0.031469 | | |
| hsa-miR-19b-1* | | 0.295688 | | 0.094976 | 3.1133 | | 0.00185 | 0.688228 | 0.031469 | | |
| hsa-miR-744* | | 0.319647 | | 0.102729 | 3.11155 | | 0.001861 | 0.692323 | 0.031469 | | |
| hsa-miR-362-3p | | 0.289345 | | 0.095532 | 3.028781 | | 0.002455 | 0.913419 | 0.039714 | | |
| hsa-miR-146b-5p | | 0.31435 | | 0.104494 | 3.008306 | | 0.002627 | 0.977275 | 0.04072 | | |
| hsa-miR-627 | | 0.325022 | | 0.111926 | 2.903893 | | 0.003686 | 1 | 0.054289 | | |
| hsa-miR-618 | | 0.288178 | | 0.100298 | 2.873226 | | 0.004063 | 1 | 0.05598 | | |
| hsa-miR-338-3p | | 0.312899 | | 0.109539 | 2.856506 | | 0.004283 | 1 | 0.056824 | | |
| hsa-miR-625* | | 0.289831 | | 0.101845 | 2.845811 | | 0.00443 | 1 | 0.056824 | | |
| hsa-miR-616 | | 0.27461 | | 0.09723 | 2.824333 | | 0.004738 | 1 | 0.057085 | | |
| hsa-miR-26b* | | 0.294758 | | 0.104412 | 2.823038 | | 0.004757 | 1 | 0.057085 | | |
| hsa-miR-28-5p | | 0.267441 | | 0.095448 | 2.801959 | | 0.005079 | 1 | 0.059047 | | |
| hsa-miR-542-5p | | 0.339258 | | 0.12192 | 2.782615 | | 0.005392 | 1 | 0.060786 | | |
| hsa-miR-148a* | | 0.300678 | | 0.108948 | 2.759823 | | 0.005783 | 1 | 0.061962 | | |
| hsa-miR-330-5p | | 0.319253 | | 0.116518 | 2.739943 | | 0.006145 | 1 | 0.061962 | | |
| hsa-miR-33a* | | 0.305032 | | 0.111367 | 2.738986 | | 0.006163 | 1 | 0.061962 | | |
| hsa-miR-505* | | 0.272137 | | 0.100268 | 2.714097 | | 0.006646 | 1 | 0.065058 | | |
| hsa-miR-215 | | 0.275709 | | 0.102282 | 2.695582 | | 0.007027 | 1 | 0.067023 | | |
| hsa-miR-149* | | 0.293464 | | 0.109277 | 2.685504 | | 0.007242 | 1 | 0.067351 | | |
| hsa-miR-221* | | 0.24959 | | 0.09528 | 2.619553 | | 0.008805 | 1 | 0.079885 | | |
| hsa-miR-361-5p | | 0.262616 | | 0.101004 | 2.600055 | | 0.009321 | 1 | 0.082556 | | |
| hsa-miR-26a-1* | | 0.296563 | | 0.115012 | 2.57855 | | 0.009922 | 1 | 0.084113 | | |
| hsa-miR-889 | | 0.279502 | | 0.10871 | 2.571072 | | 0.010138 | 1 | 0.084113 | | |
| hsa-miR-650 | | 0.27523 | | 0.107419 | 2.562203 | | 0.010401 | 1 | 0.084113 | | |
| hsa-miR-597 | | 0.259246 | | 0.101668 | 2.549933 | | 0.010774 | 1 | 0.085278 | | |
| hsa-let-7f-2* | | 0.288995 | | 0.113802 | 2.539452 | | 0.011103 | 1 | 0.086045 | | |
| hsa-miR-483-5p | | 0.227317 | | 0.090665 | 2.507215 | | 0.012169 | 1 | 0.089023 | | |
| hsa-miR-181a | | 0.239275 | | 0.0956 | 2.50288 | | 0.012319 | 1 | 0.089023 | | |
| hsa-miR-598 | | 0.278553 | | 0.111631 | 2.495294 | | 0.012585 | 1 | 0.089023 | | |
| hsa-miR-339-3p | | 0.261934 | | 0.105087 | 2.492537 | | 0.012683 | 1 | 0.089023 | | |
| hsa-miR-192* | | 0.267355 | | 0.107866 | 2.478579 | | 0.013191 | 1 | 0.089674 | | |
| hsa-miR-503 | | 0.266963 | | 0.107787 | 2.476757 | | 0.013258 | 1 | 0.089674 | | |
| hsa-miR-199b-5p | | 0.246558 | | 0.10023 | 2.459929 | | 0.013896 | 1 | 0.092312 | | |
| hsa-miR-197 | | 0.250143 | | 0.103148 | 2.425084 | | 0.015305 | 1 | 0.099884 | | |
| hsa-miR-331-5p | | 0.237963 | | 0.099804 | 2.384297 | | 0.017112 | 1 | 0.109751 | | |
| hsa-miR-24 | | 0.251074 | | 0.107308 | 2.339748 | | 0.019297 | 1 | 0.121668 | | |
| hsa-miR-29c* | | 0.235498 | | 0.100923 | 2.333456 | | 0.019624 | 1 | 0.12167 | | |
| hsa-miR-106b* | | 0.218017 | | 0.093977 | 2.319903 | | 0.020346 | 1 | 0.124078 | | |
| hsa-miR-181c* | | 0.217344 | | 0.096118 | 2.261222 | | 0.023746 | 1 | 0.142473 | | |
| hsa-miR-139-3p | | 0.239279 | | 0.106762 | 2.241226 | | 0.025011 | 1 | 0.147687 | | |
| hsa-miR-182* | | 0.231538 | | 0.104916 | 2.206897 | | 0.027321 | 1 | 0.157263 | | |
| hsa-miR-183* | | 0.230239 | | 0.104453 | 2.204243 | | 0.027507 | 1 | 0.157263 | | |
| hsa-miR-551a | | 0.221204 | | 0.101158 | 2.186724 | | 0.028763 | 1 | 0.159697 | | |
| hsa-miR-379* | | 0.219947 | | 0.101231 | 2.172735 | | 0.0298 | 1 | 0.163025 | | |
| hsa-miR-376a | | 0.219004 | | 0.102083 | 2.145347 | | 0.031925 | 1 | 0.168076 | | |
| hsa-miR-935 | | 0.201029 | | 0.093777 | 2.143684 | | 0.032058 | 1 | 0.168076 | | |
| hsa-miR-511 | | 0.199129 | | 0.093194 | 2.13671 | | 0.032622 | 1 | 0.168076 | | |
| hsa-miR-27b | | 0.217476 | | 0.1018 | 2.136307 | | 0.032654 | 1 | 0.168076 | | |
| hsa-miR-886-5p | | 0.199072 | | 0.094108 | 2.115361 | | 0.034399 | 1 | 0.168076 | | |
| hsa-miR-411* | | 0.243408 | | 0.115749 | 2.102883 | | 0.035476 | 1 | 0.168076 | | |
| hsa-miR-550 | | 0.206432 | | 0.098235 | 2.101405 | | 0.035605 | 1 | 0.168076 | | |
| hsa-miR-148b | | 0.209807 | | 0.09985 | 2.101211 | | 0.035622 | 1 | 0.168076 | | |
| hsa-miR-502-5p | | 0.191937 | | 0.091381 | 2.100401 | | 0.035694 | 1 | 0.168076 | | |
| hsa-miR-494 | | 0.223655 | | 0.109044 | 2.05105 | | 0.040262 | 1 | 0.182652 | | |
| hsa-miR-30d* | | 0.238949 | | 0.118007 | 2.024875 | | 0.04288 | 1 | 0.192186 | | |
| hsa-miR-625 | | 0.184612 | | 0.09332 | 1.978265 | | 0.047899 | 1 | 0.209628 | | |
| hsa-miR-148b* | | 0.200003 | | 0.101501 | 1.970454 | | 0.048786 | 1 | 0.211029 | | |
| hsa-miR-199a-3p | | 0.180628 | | 0.092129 | 1.960605 | | 0.049925 | 1 | 0.213473 | | |
| hsa-miR-328 | | -0.22133 | | 0.109588 | -2.01969 | | 0.043416 | 1 | 0.192269 | | |
| hsa-miR-636 | | -0.20771 | | 0.100356 | -2.06976 | | 0.038475 | 1 | 0.176699 | | |
| hsa-miR-652 | | -0.20752 | | 0.099584 | -2.08391 | | 0.037169 | 1 | 0.172835 | | |
| hsa-miR-505 | | -0.20773 | | 0.098796 | -2.10265 | | 0.035496 | 1 | 0.168076 | | |
| hsa-miR-484 | | -0.19745 | | 0.093368 | -2.11478 | | 0.034448 | 1 | 0.168076 | | |
| hsa-miR-144* | | -0.2052 | | 0.093327 | -2.19867 | | 0.027902 | 1 | 0.157263 | | |
| hsa-miR-589* | | -0.24045 | | 0.096396 | -2.49437 | | 0.012618 | 1 | 0.089023 | | |
| hsa-miR-532-3p | | -0.30774 | | 0.119772 | -2.56939 | | 0.010188 | 1 | 0.084113 | | |
| hsa-miR-150 | | -0.25113 | | 0.091492 | -2.74488 | | 0.006053 | 1 | 0.061962 | | |
| hsa-miR-92a-1* | | -0.31987 | | 0.1105 | -2.89477 | | 0.003794 | 1 | 0.054289 | | |
| hsa-miR-31 | | -0.33987 | | 0.096425 | -3.52473 | | 0.000424 | 0.157697 | 0.017522 | | |
| hsa-miR-342-5p | | -0.35635 | | 0.096353 | -3.69839 | | 0.000217 | 0.080714 | 0.011531 | | |
| **hsa-miR-324-3p** | | **-0.4528** | | **0.10431** | **-4.3409** | | **1.42E-05** | **0.005279** | **0.00176** | | |

# Table S4

Table S4 miRNAs prognostic for OS in patients with metastatic colorectal cancer in the subset of patients with KRAS and BRAF double WT. In bold those with Bonferroni *P*-value < 0.05 *est: estimate, se: standard error, P Wald: P-value from Wald test, P Bonferroni: P-value adjusted by the Bonferroni method, P*-FDR**:** *P-value from FDR (False Discovery Rate).*

| **miRNA** | **est** | **se** | **Z** | ***P* Wald** | ***P* Bonferroni** | ***P*-FDR** |
| --- | --- | --- | --- | --- | --- | --- |
| **hsa-miR-345** | **0.544785** | **0.134346** | **4.055095** | **5.01E-05** | **0.018642** | **0.018642** |
| hsa-miR-943 | 0.537436 | 0.145613 | 3.690859 | 0.000223 | 0.083141 | 0.041571 |
| hsa-miR-628-5p | 0.55369 | 0.161291 | 3.432858 | 0.000597 | 0.222179 | 0.07406 |
| hsa-miR-34a* | 0.43246 | 0.129403 | 3.341954 | 0.000832 | 0.30947 | 0.077367 |
| hsa-miR-26b* | 0.458824 | 0.145832 | 3.146245 | 0.001654 | 0.615218 | 0.099192 |
| hsa-miR-936 | 0.422125 | 0.134993 | 3.127013 | 0.001766 | 0.656922 | 0.099192 |
| hsa-miR-143 | 0.46786 | 0.150404 | 3.110688 | 0.001867 | 0.694346 | 0.099192 |
| hsa-miR-210 | 0.367385 | 0.12412 | 2.959926 | 0.003077 | 1 | 0.126668 |
| hsa-miR-19b-1* | 0.349911 | 0.118372 | 2.956029 | 0.003116 | 1 | 0.126668 |
| hsa-miR-886-3p | 0.356617 | 0.123974 | 2.876549 | 0.00402 | 1 | 0.126668 |
| hsa-miR-194 | 0.379247 | 0.132324 | 2.866048 | 0.004156 | 1 | 0.126668 |
| hsa-miR-625* | 0.356632 | 0.12582 | 2.834464 | 0.00459 | 1 | 0.126668 |
| hsa-miR-338-3p | 0.382965 | 0.135689 | 2.822366 | 0.004767 | 1 | 0.126668 |
| hsa-miR-145 | 0.370997 | 0.133641 | 2.776072 | 0.005502 | 1 | 0.128506 |
| hsa-miR-7 | 0.419441 | 0.151172 | 2.77459 | 0.005527 | 1 | 0.128506 |
| hsa-miR-616 | 0.362487 | 0.133017 | 2.725112 | 0.006428 | 1 | 0.132845 |
| hsa-miR-29a | 0.340286 | 0.126359 | 2.69301 | 0.007081 | 1 | 0.13699 |
| hsa-miR-330-3p | 0.333644 | 0.1245 | 2.679872 | 0.007365 | 1 | 0.13699 |
| hsa-miR-145* | 0.345965 | 0.130265 | 2.655851 | 0.007911 | 1 | 0.140135 |
| hsa-miR-744* | 0.368358 | 0.141681 | 2.599914 | 0.009325 | 1 | 0.148108 |
| hsa-miR-192 | 0.309396 | 0.119218 | 2.595226 | 0.009453 | 1 | 0.148108 |
| hsa-miR-550 | 0.323416 | 0.125067 | 2.58595 | 0.009711 | 1 | 0.148108 |
| hsa-miR-627 | 0.345083 | 0.134997 | 2.556226 | 0.010581 | 1 | 0.148108 |
| hsa-miR-589 | 0.361611 | 0.142674 | 2.534534 | 0.01126 | 1 | 0.148108 |
| hsa-miR-889 | 0.369305 | 0.145854 | 2.532013 | 0.011341 | 1 | 0.148108 |
| hsa-miR-551a | 0.333037 | 0.131858 | 2.525724 | 0.011546 | 1 | 0.148108 |
| hsa-let-7g | 0.323004 | 0.131402 | 2.458141 | 0.013966 | 1 | 0.173176 |
| hsa-miR-330-5p | 0.35412 | 0.146598 | 2.415584 | 0.01571 | 1 | 0.177095 |
| hsa-miR-221* | 0.29297 | 0.122526 | 2.391091 | 0.016798 | 1 | 0.183794 |
| hsa-miR-10b | 0.36525 | 0.156688 | 2.331069 | 0.01975 | 1 | 0.205643 |
| hsa-miR-26a-1* | 0.333144 | 0.144458 | 2.306167 | 0.021101 | 1 | 0.205643 |
| hsa-miR-192* | 0.323532 | 0.140928 | 2.295725 | 0.021692 | 1 | 0.205643 |
| hsa-miR-148a* | 0.325436 | 0.141805 | 2.29496 | 0.021735 | 1 | 0.205643 |
| hsa-miR-362-3p | 0.28957 | 0.126954 | 2.280902 | 0.022554 | 1 | 0.205643 |
| hsa-miR-33a* | 0.329826 | 0.144722 | 2.279036 | 0.022665 | 1 | 0.205643 |
| hsa-miR-34a | 0.278307 | 0.122623 | 2.269614 | 0.023231 | 1 | 0.20576 |
| hsa-miR-27a* | 0.310633 | 0.139506 | 2.226664 | 0.02597 | 1 | 0.220648 |
| hsa-miR-30d* | 0.359881 | 0.161763 | 2.224748 | 0.026098 | 1 | 0.220648 |
| hsa-miR-28-5p | 0.271994 | 0.123735 | 2.1982 | 0.027935 | 1 | 0.230928 |
| hsa-miR-215 | 0.269026 | 0.124682 | 2.157694 | 0.030952 | 1 | 0.250305 |
| hsa-miR-29c | 0.305648 | 0.142553 | 2.14411 | 0.032024 | 1 | 0.253467 |
| hsa-miR-30a* | 0.231441 | 0.108672 | 2.129712 | 0.033195 | 1 | 0.253565 |
| hsa-miR-186* | 0.240472 | 0.113409 | 2.120397 | 0.033973 | 1 | 0.253565 |
| hsa-miR-650 | 0.286362 | 0.135133 | 2.119108 | 0.034081 | 1 | 0.253565 |
| hsa-miR-148b* | 0.28908 | 0.137479 | 2.102731 | 0.035489 | 1 | 0.257156 |
| hsa-miR-197 | 0.275397 | 0.131296 | 2.097532 | 0.035946 | 1 | 0.257156 |
| hsa-miR-181c* | 0.255847 | 0.122688 | 2.085351 | 0.037037 | 1 | 0.25799 |
| hsa-miR-505* | 0.262341 | 0.126109 | 2.080265 | 0.037501 | 1 | 0.25799 |
| hsa-miR-181a | 0.273867 | 0.132092 | 2.073307 | 0.038144 | 1 | 0.25799 |
| hsa-miR-212 | 0.245341 | 0.119424 | 2.054361 | 0.039941 | 1 | 0.265321 |
| hsa-miR-149* | 0.276517 | 0.135948 | 2.033987 | 0.041953 | 1 | 0.273798 |
| hsa-miR-339-3p | 0.248166 | 0.123612 | 2.007631 | 0.044683 | 1 | 0.281418 |
| hsa-miR-542-5p | 0.304503 | 0.151741 | 2.00673 | 0.044778 | 1 | 0.281418 |
| hsa-let-7f-2* | 0.289236 | 0.144544 | 2.001022 | 0.04539 | 1 | 0.281418 |
| hsa-miR-938 | 0.289437 | 0.145675 | 1.986871 | 0.046937 | 1 | 0.281973 |
| hsa-miR-935 | 0.228469 | 0.11502 | 1.986341 | 0.046996 | 1 | 0.281973 |
| hsa-miR-618 | 0.248694 | 0.126783 | 1.96157 | 0.049813 | 1 | 0.294131 |
| hsa-miR-95 | -0.3095 | 0.133236 | -2.32291 | 0.020184 | 1 | 0.205643 |
| hsa-miR-342-5p | -0.26747 | 0.110163 | -2.42797 | 0.015183 | 1 | 0.176508 |
| hsa-miR-150 | -0.28345 | 0.11617 | -2.43992 | 0.014691 | 1 | 0.176288 |
| hsa-miR-92a-1* | -0.34737 | 0.13729 | -2.53017 | 0.011401 | 1 | 0.148108 |
| hsa-miR-324-3p | -0.33805 | 0.123824 | -2.73012 | 0.006331 | 1 | 0.132845 |
| hsa-miR-31 | -0.35783 | 0.12398 | -2.88619 | 0.003899 | 1 | 0.126668 |

# Table S5

Table S5 miRNAs prognostic for OS in patients with metastatic colorectal cancer in the subset of patients with PI3KCA wt. In bold those with Bonferroni *P*-value < 0.05. *est: estimate, se: standard error, P Wald: P-value from Wald test, P Bonferroni: P-value adjusted by the Bonferroni method, P*-FDR**:** *P-value from FDR (False Discovery Rate).*

| **miRNA** | **est** | **se** | **Z** | ***P* Wald** | ***P* Bonferroni** | ***P*-FDR** |
| --- | --- | --- | --- | --- | --- | --- |
| **hsa-miR-345** | **0.637387** | **0.115518** | **5.51766** | **3.44E-08** | **1.28E-05** | **1.28E-05** |
| **hsa-miR-30a*** | **0.512023** | **0.115214** | **4.444103** | **8.83E-06** | **0.003283** | **0.001642** |
| **hsa-miR-143** | **0.531895** | **0.126237** | **4.213467** | **2.51E-05** | **0.009355** | **0.002163** |
| **hsa-miR-145*** | **0.479055** | **0.114588** | **4.180663** | **2.91E-05** | **0.010813** | **0.002163** |
| **hsa-miR-145** | **0.479461** | **0.11773** | **4.07254** | **4.65E-05** | **0.017299** | **0.002883** |
| **hsa-miR-943** | **0.454014** | **0.113594** | **3.99681** | **6.42E-05** | **0.023883** | **0.003412** |
| **hsa-miR-936** | **0.498111** | **0.129418** | **3.848859** | **0.000119** | **0.044145** | **0.005518** |
| hsa-miR-34a* | 0.419714 | 0.112532 | 3.729744 | 0.000192 | 0.071303 | 0.007923 |
| hsa-miR-744* | 0.413657 | 0.114823 | 3.602545 | 0.000315 | 0.117223 | 0.010657 |
| hsa-miR-29a | 0.385771 | 0.108163 | 3.566582 | 0.000362 | 0.134541 | 0.011212 |
| hsa-miR-192* | 0.409879 | 0.11985 | 3.419917 | 0.000626 | 0.233022 | 0.017925 |
| hsa-miR-7 | 0.412157 | 0.125537 | 3.283155 | 0.001027 | 0.381866 | 0.025458 |
| hsa-miR-194 | 0.350495 | 0.110299 | 3.177688 | 0.001485 | 0.552251 | 0.034516 |
| hsa-miR-628-5p | 0.441742 | 0.141478 | 3.122347 | 0.001794 | 0.667425 | 0.036773 |
| hsa-miR-330-3p | 0.332025 | 0.106639 | 3.113544 | 0.001849 | 0.68766 | 0.036773 |
| hsa-miR-146b-5p | 0.36439 | 0.117272 | 3.107209 | 0.001889 | 0.702569 | 0.036773 |
| hsa-miR-192 | 0.345188 | 0.111579 | 3.093659 | 0.001977 | 0.735461 | 0.036773 |
| hsa-miR-28-5p | 0.304387 | 0.100498 | 3.028793 | 0.002455 | 0.913383 | 0.041422 |
| hsa-miR-26b* | 0.339754 | 0.112415 | 3.022326 | 0.002508 | 0.933127 | 0.041422 |
| hsa-miR-886-3p | 0.331645 | 0.110291 | 3.00701 | 0.002638 | 0.981453 | 0.041422 |
| hsa-miR-618 | 0.417924 | 0.13968 | 2.992018 | 0.002771 | 1 | 0.041422 |
| hsa-miR-362-3p | 0.295121 | 0.099232 | 2.974051 | 0.002939 | 1 | 0.041422 |
| hsa-miR-935 | 0.309895 | 0.104685 | 2.960256 | 0.003074 | 1 | 0.041422 |
| hsa-miR-616 | 0.316424 | 0.106996 | 2.957336 | 0.003103 | 1 | 0.041422 |
| hsa-miR-148a* | 0.340174 | 0.115567 | 2.943536 | 0.003245 | 1 | 0.041422 |
| hsa-miR-505* | 0.3195 | 0.108876 | 2.934537 | 0.00334 | 1 | 0.041422 |
| hsa-miR-331-5p | 0.313438 | 0.109364 | 2.866015 | 0.004157 | 1 | 0.049881 |
| hsa-miR-34a | 0.286167 | 0.100916 | 2.835703 | 0.004572 | 1 | 0.052195 |
| hsa-miR-889 | 0.337894 | 0.119465 | 2.828386 | 0.004678 | 1 | 0.052195 |
| hsa-miR-26a-1* | 0.369523 | 0.130937 | 2.822137 | 0.00477 | 1 | 0.052195 |
| hsa-miR-210 | 0.304159 | 0.109821 | 2.769578 | 0.005613 | 1 | 0.058 |
| hsa-miR-361-5p | 0.313346 | 0.113612 | 2.758045 | 0.005815 | 1 | 0.058462 |
| hsa-miR-215 | 0.306337 | 0.11186 | 2.738566 | 0.006171 | 1 | 0.060409 |
| hsa-miR-19b-1* | 0.268095 | 0.098712 | 2.715934 | 0.006609 | 1 | 0.063039 |
| hsa-miR-33a* | 0.311859 | 0.116364 | 2.680025 | 0.007362 | 1 | 0.068055 |
| hsa-miR-197 | 0.309598 | 0.11582 | 2.673085 | 0.007516 | 1 | 0.068055 |
| hsa-miR-199b-5p | 0.281058 | 0.105512 | 2.663746 | 0.007728 | 1 | 0.068055 |
| hsa-miR-338-3p | 0.315706 | 0.119027 | 2.652394 | 0.007992 | 1 | 0.068055 |
| hsa-miR-181c* | 0.280204 | 0.105738 | 2.649985 | 0.00805 | 1 | 0.068055 |
| hsa-miR-181a | 0.267356 | 0.102789 | 2.601017 | 0.009295 | 1 | 0.076837 |
| hsa-miR-30e | 0.284314 | 0.110003 | 2.584592 | 0.009749 | 1 | 0.078843 |
| hsa-miR-511 | 0.266996 | 0.104584 | 2.552931 | 0.010682 | 1 | 0.084547 |
| hsa-miR-625* | 0.28164 | 0.110789 | 2.542142 | 0.011018 | 1 | 0.084796 |
| hsa-miR-183* | 0.294149 | 0.115927 | 2.537355 | 0.011169 | 1 | 0.084796 |
| hsa-miR-650 | 0.294453 | 0.117505 | 2.505878 | 0.012215 | 1 | 0.090021 |
| hsa-let-7f-2* | 0.299836 | 0.119828 | 2.502225 | 0.012342 | 1 | 0.090021 |
| hsa-miR-551a | 0.263055 | 0.108083 | 2.433815 | 0.014941 | 1 | 0.106883 |
| hsa-let-7f-1* | 0.269087 | 0.112223 | 2.397787 | 0.016494 | 1 | 0.115772 |
| hsa-miR-27b | 0.260558 | 0.110431 | 2.359468 | 0.018301 | 1 | 0.12492 |
| hsa-miR-24 | 0.275592 | 0.117351 | 2.348442 | 0.018852 | 1 | 0.12492 |
| hsa-miR-149* | 0.272643 | 0.116462 | 2.341054 | 0.019229 | 1 | 0.12492 |
| hsa-miR-221* | 0.244618 | 0.104609 | 2.338404 | 0.019366 | 1 | 0.12492 |
| hsa-miR-106b* | 0.232249 | 0.09941 | 2.336276 | 0.019477 | 1 | 0.12492 |
| hsa-miR-502-5p | 0.225063 | 0.096664 | 2.328306 | 0.019896 | 1 | 0.125445 |
| hsa-miR-339-3p | 0.267515 | 0.115536 | 2.315431 | 0.020589 | 1 | 0.127654 |
| hsa-miR-127-5p | 0.258083 | 0.112578 | 2.292483 | 0.021878 | 1 | 0.131267 |
| hsa-miR-542-5p | 0.287496 | 0.126573 | 2.271391 | 0.023123 | 1 | 0.136538 |
| hsa-miR-20b* | 0.258663 | 0.11485 | 2.252177 | 0.024311 | 1 | 0.141308 |
| hsa-miR-340* | 0.260732 | 0.117252 | 2.22369 | 0.026169 | 1 | 0.149769 |
| hsa-miR-627 | 0.267605 | 0.120709 | 2.216938 | 0.026627 | 1 | 0.150081 |
| hsa-miR-503 | 0.251151 | 0.114553 | 2.192443 | 0.028348 | 1 | 0.155078 |
| hsa-miR-330-5p | 0.265765 | 0.125016 | 2.125836 | 0.033517 | 1 | 0.179992 |
| hsa-miR-199a-3p | 0.205853 | 0.097026 | 2.121622 | 0.03387 | 1 | 0.179992 |
| hsa-miR-148b* | 0.228184 | 0.107878 | 2.115212 | 0.034412 | 1 | 0.180299 |
| hsa-miR-550 | 0.214389 | 0.102369 | 2.094284 | 0.036235 | 1 | 0.187213 |
| hsa-miR-26a | 0.258131 | 0.124107 | 2.079901 | 0.037535 | 1 | 0.191272 |
| hsa-miR-597 | 0.227981 | 0.111639 | 2.042135 | 0.041138 | 1 | 0.206803 |
| hsa-miR-411* | 0.258773 | 0.127498 | 2.029627 | 0.042394 | 1 | 0.208918 |
| hsa-miR-139-5p | 0.272529 | 0.134462 | 2.026807 | 0.042682 | 1 | 0.208918 |
| hsa-miR-650 | 0.248519 | 0.123365 | 2.014504 | 0.043957 | 1 | 0.209973 |
| hsa-miR-29c* | 0.213578 | 0.107497 | 1.986825 | 0.046942 | 1 | 0.209973 |
| hsa-miR-483-5p | 0.194312 | 0.098524 | 1.972231 | 0.048583 | 1 | 0.209973 |
| hsa-miR-139-3p | 0.228328 | 0.115826 | 1.97131 | 0.048688 | 1 | 0.209973 |
| hsa-miR-636 | -0.2109 | 0.107184 | -1.96766 | 0.049107 | 1 | 0.209973 |
| hsa-miR-144* | -0.20898 | 0.105892 | -1.97351 | 0.048438 | 1 | 0.209973 |
| hsa-miR-191 | -0.19078 | 0.096216 | -1.98278 | 0.047392 | 1 | 0.209973 |
| hsa-miR-484 | -0.19501 | 0.097934 | -1.99122 | 0.046457 | 1 | 0.209973 |
| hsa-let-7c | -0.22402 | 0.112499 | -1.99133 | 0.046444 | 1 | 0.209973 |
| hsa-miR-874 | -0.22493 | 0.112498 | -1.99942 | 0.045563 | 1 | 0.209973 |
| hsa-miR-501-5p | -0.20469 | 0.102119 | -2.0044 | 0.045027 | 1 | 0.209973 |
| hsa-miR-652 | -0.22872 | 0.103914 | -2.20107 | 0.027731 | 1 | 0.153969 |
| hsa-miR-589* | -0.23894 | 0.104114 | -2.29503 | 0.021731 | 1 | 0.131267 |
| hsa-miR-92a-1* | -0.34077 | 0.122574 | -2.78015 | 0.005433 | 1 | 0.057749 |
| hsa-miR-150 | -0.31599 | 0.107094 | -2.95062 | 0.003171 | 1 | 0.041422 |
| hsa-miR-31 | -0.35952 | 0.107719 | -3.33756 | 0.000845 | 0.314407 | 0.022458 |
| **hsa-miR-342-5p** | **-0.39071** | **0.106082** | **-3.68313** | **0.00023** | **0.085704** | **0.00857** |
| **hsa-miR-324-3p** | **-0.4797** | **0.11143** | **-4.30493** | **1.67E-05** | **0.006214** | **0.002071** |

# Table S6

Table S6 miRNAs prognostic for OS in patients with metastatic colorectal cancer in the subset of patients with KRAS WT and PI3KCA WT. In bold those with Bonferroni *P*-value < 0.05. *est: estimate, se: standard error, P Wald: P-value from Wald test, P Bonferroni: P-value adjusted by the Bonferroni method, P*-FDR**:** *P-value from FDR (False Discovery Rate).*

| **miRNA** | **est** | **se** | **Z** | ***P* Wald** | ***P* Bonferroni** | ***P*-FDR** |
| --- | --- | --- | --- | --- | --- | --- |
| **hsa-miR-345** | **0.685481** | **0.148645** | **4.611544** | **4.00E-06** | **0.001487** | **0.001487** |
| **hsa-miR-943** | **0.634449** | **0.152634** | **4.156666** | **3.23E-05** | **0.012013** | **0.006006** |
| **hsa-miR-145** | **0.609893** | **0.156627** | **3.89391** | **9.86E-05** | **0.036695** | **0.010015** |
| **hsa-miR-30a*** | **0.561808** | **0.145073** | **3.872587** | **0.000108** | **0.040059** | **0.010015** |
| hsa-miR-145* | 0.595521 | 0.156201 | 3.81253 | 0.000138 | 0.051169 | 0.010234 |
| hsa-miR-192* | 0.572909 | 0.155983 | 3.672897 | 0.00024 | 0.089212 | 0.012895 |
| hsa-miR-628-5p | 0.631467 | 0.172698 | 3.656483 | 0.000256 | 0.09512 | 0.012895 |
| hsa-miR-34a* | 0.516806 | 0.142732 | 3.620823 | 0.000294 | 0.109244 | 0.012895 |
| hsa-miR-936 | 0.585427 | 0.162386 | 3.605153 | 0.000312 | 0.116053 | 0.012895 |
| hsa-miR-744* | 0.584472 | 0.16536 | 3.53454 | 0.000408 | 0.151957 | 0.014131 |
| hsa-miR-26b* | 0.552213 | 0.156498 | 3.528553 | 0.000418 | 0.155436 | 0.014131 |
| hsa-miR-616 | 0.516115 | 0.149982 | 3.441178 | 0.000579 | 0.215458 | 0.017927 |
| hsa-miR-143 | 0.552044 | 0.161422 | 3.419883 | 0.000626 | 0.23305 | 0.017927 |
| hsa-miR-330-3p | 0.437139 | 0.138294 | 3.160934 | 0.001573 | 0.585023 | 0.039002 |
| hsa-miR-589 | 0.480924 | 0.155906 | 3.084711 | 0.002038 | 0.75795 | 0.041829 |
| hsa-miR-551a | 0.432288 | 0.140319 | 3.080751 | 0.002065 | 0.768104 | 0.041829 |
| hsa-miR-338-3p | 0.458376 | 0.149762 | 3.060697 | 0.002208 | 0.821458 | 0.041829 |
| hsa-miR-30e | 0.450637 | 0.14803 | 3.044231 | 0.002333 | 0.867787 | 0.041829 |
| hsa-miR-7 | 0.490805 | 0.161419 | 3.040571 | 0.002361 | 0.878405 | 0.041829 |
| hsa-miR-148a* | 0.461921 | 0.153532 | 3.008624 | 0.002624 | 0.976252 | 0.044375 |
| hsa-miR-886-3p | 0.407899 | 0.139289 | 2.928438 | 0.003407 | 1 | 0.054909 |
| hsa-miR-19b-1* | 0.351803 | 0.120635 | 2.916264 | 0.003543 | 1 | 0.054909 |
| hsa-miR-210 | 0.384142 | 0.133192 | 2.88411 | 0.003925 | 1 | 0.05745 |
| hsa-miR-505* | 0.38317 | 0.133741 | 2.865025 | 0.00417 | 1 | 0.05745 |
| hsa-miR-194 | 0.383201 | 0.135572 | 2.826538 | 0.004705 | 1 | 0.062515 |
| hsa-miR-181a | 0.408621 | 0.146531 | 2.788631 | 0.005293 | 1 | 0.067898 |
| hsa-miR-550 | 0.342815 | 0.124605 | 2.751221 | 0.005937 | 1 | 0.073623 |
| hsa-miR-29a | 0.349672 | 0.128841 | 2.713978 | 0.006648 | 1 | 0.079777 |
| hsa-miR-362-3p | 0.343022 | 0.127345 | 2.693649 | 0.007067 | 1 | 0.082159 |
| hsa-miR-28-5p | 0.337321 | 0.127093 | 2.654119 | 0.007952 | 1 | 0.088854 |
| hsa-miR-181c* | 0.354009 | 0.135883 | 2.605241 | 0.009181 | 1 | 0.095079 |
| hsa-miR-29c | 0.393889 | 0.151235 | 2.604489 | 0.009201 | 1 | 0.095079 |
| hsa-miR-26a-1* | 0.424705 | 0.165586 | 2.564866 | 0.010322 | 1 | 0.103774 |
| hsa-miR-340* | 0.421323 | 0.168742 | 2.496845 | 0.01253 | 1 | 0.122666 |
| hsa-miR-935 | 0.318621 | 0.12855 | 2.478566 | 0.013191 | 1 | 0.123308 |
| hsa-miR-33a* | 0.361911 | 0.146124 | 2.476738 | 0.013259 | 1 | 0.123308 |
| hsa-miR-127-5p | 0.34183 | 0.139509 | 2.450231 | 0.014276 | 1 | 0.129533 |
| hsa-miR-148b* | 0.342971 | 0.141644 | 2.421361 | 0.015463 | 1 | 0.136303 |
| hsa-miR-618 | 0.451438 | 0.187859 | 2.403073 | 0.016258 | 1 | 0.136303 |
| hsa-miR-192 | 0.302457 | 0.126394 | 2.392966 | 0.016713 | 1 | 0.136303 |
| hsa-miR-938 | 0.378145 | 0.158343 | 2.388139 | 0.016934 | 1 | 0.136303 |
| hsa-miR-625* | 0.324627 | 0.136057 | 2.385972 | 0.017034 | 1 | 0.136303 |
| hsa-miR-215 | 0.323248 | 0.135707 | 2.381953 | 0.017221 | 1 | 0.136303 |
| hsa-miR-30d* | 0.416703 | 0.178457 | 2.33504 | 0.019541 | 1 | 0.151446 |
| hsa-miR-889 | 0.357025 | 0.154866 | 2.305386 | 0.021145 | 1 | 0.159668 |
| hsa-miR-183* | 0.341615 | 0.149028 | 2.292292 | 0.021889 | 1 | 0.159668 |
| hsa-let-7f-2* | 0.343741 | 0.149957 | 2.292271 | 0.02189 | 1 | 0.159668 |
| hsa-miR-361-5p | 0.336383 | 0.151394 | 2.221908 | 0.026289 | 1 | 0.188071 |
| hsa-miR-199b-5p | 0.297651 | 0.134778 | 2.208459 | 0.027212 | 1 | 0.190999 |
| hsa-miR-502-5p | 0.260285 | 0.1186 | 2.194643 | 0.028189 | 1 | 0.194193 |
| hsa-miR-197 | 0.314884 | 0.144106 | 2.185079 | 0.028883 | 1 | 0.195354 |
| hsa-miR-330-5p | 0.332277 | 0.153938 | 2.158508 | 0.030888 | 1 | 0.204697 |
| hsa-miR-221* | 0.297943 | 0.138423 | 2.152413 | 0.031365 | 1 | 0.204697 |
| hsa-miR-650 | 0.312047 | 0.145846 | 2.139571 | 0.032389 | 1 | 0.207739 |
| hsa-miR-627 | 0.30234 | 0.142225 | 2.125792 | 0.033521 | 1 | 0.21135 |
| hsa-miR-331-5p | 0.266779 | 0.127038 | 2.099995 | 0.035729 | 1 | 0.221522 |
| hsa-miR-146b-5p | 0.290321 | 0.142827 | 2.032672 | 0.042086 | 1 | 0.24341 |
| hsa-miR-212 | 0.257537 | 0.126973 | 2.028284 | 0.042531 | 1 | 0.24341 |
| hsa-miR-27a* | 0.301844 | 0.151816 | 1.988217 | 0.046788 | 1 | 0.259856 |
| hsa-miR-542-5p | 0.305875 | 0.154195 | 1.983684 | 0.047291 | 1 | 0.259856 |
| hsa-miR-17 | -0.24081 | 0.12151 | -1.98181 | 0.047501 | 1 | 0.259856 |
| hsa-miR-95 | -0.29583 | 0.143131 | -2.06684 | 0.038749 | 1 | 0.228803 |
| hsa-miR-92a-1* | -0.30845 | 0.147907 | -2.08542 | 0.037031 | 1 | 0.222188 |
| hsa-miR-769-3p | -0.29608 | 0.141771 | -2.08845 | 0.036758 | 1 | 0.222188 |
| hsa-miR-342-5p | -0.34371 | 0.129849 | -2.64699 | 0.008121 | 1 | 0.088854 |
| hsa-miR-324-3p | -0.38955 | 0.135686 | -2.871 | 0.004092 | 1 | 0.05745 |
| hsa-miR-150 | -0.41355 | 0.133196 | -3.10484 | 0.001904 | 0.708212 | 0.041829 |
| hsa-miR-31 | -0.43241 | 0.136351 | -3.17129 | 0.001518 | 0.564551 | 0.039002 |

# Figure S5.

Volcano plots showing Z score as calculated by Cox regression analysis and –log10 of the unadjusted p value.


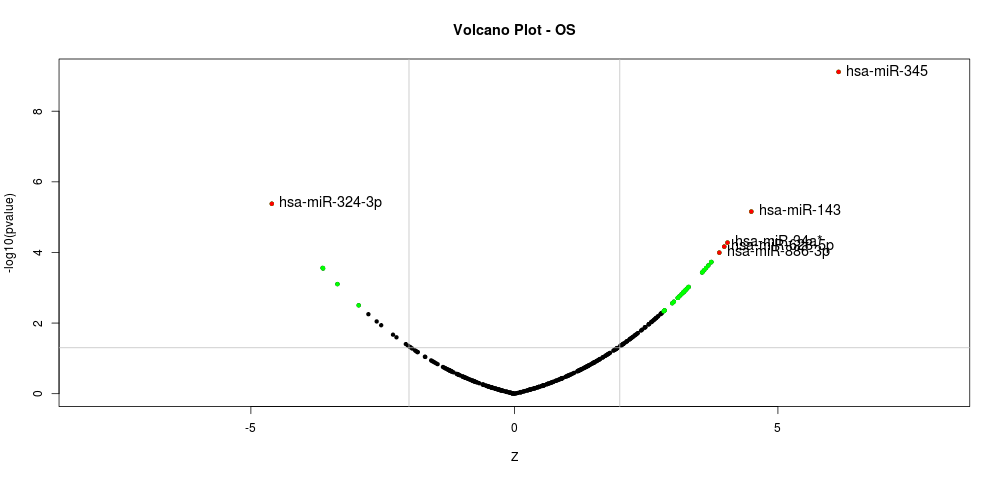


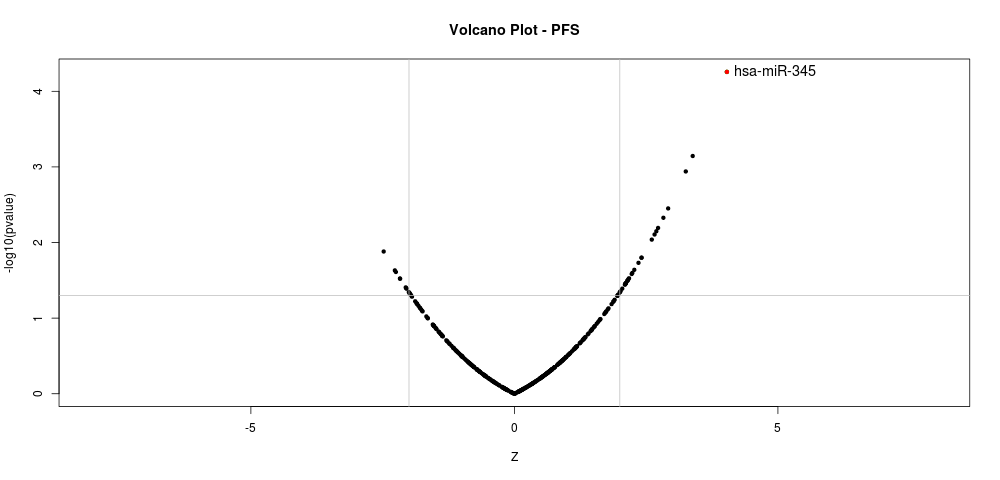


# Figure S6

Plots (OS and PFS) of the concordance probabilities calculated on the bootstrap statistics. Red dots have been drawn for miR-21, miR-345, miR-143, miR-34a*, miR-628-5p miR-886-3p and miR-324-3p.


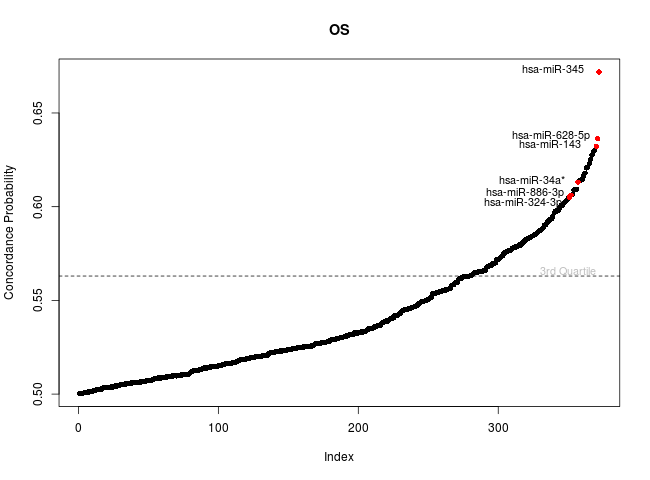


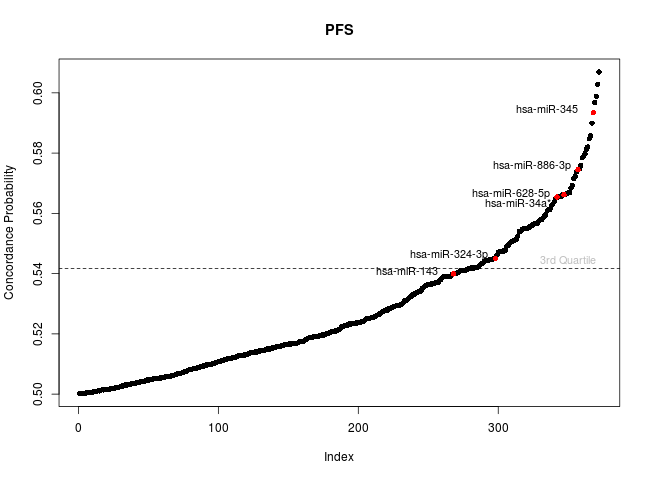

Supplement: File S1 — Contains the following files: Figure S1, Figure S2, Figure S3, Figure S4, Table S1, Table S2, Table S3, Table S4, Table S5, Table S6, Figure S5, Figure S6. (DOCX) [file pone.0099886.s001.docx]
